# An Encaustic without Wax: Microanalytical Binder Characterization of Diego Rivera’s First Mural, Experimental Reproduction, and Identification of Degradation Products

**DOI:** 10.1021/acsomega.6c00983

**Published:** 2026-07-15

**Authors:** Pablo Aguilar-Rodríguez, Joel Landa-Huerta, Angel Santiago-Jiménez, Sandra Zetina, Araceli Peña-Álvarez, Mayra León-Santiago, Nuria Esturau-Escofet

**Affiliations:** † Instituto de Química, 7180 Universidad Nacional Autónoma de México, Ciudad de México 04510, Mexico; ‡ Instituto de Investigaciones Estéticas, Universidad Nacional Autónoma de México, Ciudad de México 04510, Mexico; § Facultad de Química, Universidad Nacional Autónoma de México, Ciudad de México 04510, Mexico; ∥ Laboratorio Nacional de Ciencias para la Investigación y la Conservación del Patrimonio Cultural (LANCIC, National Science Laboratory for Research and Conservation of Cultural Heritage), Ciudad de México 04510, Mexico; 5 Departamento de Fisiología, Biofísica y Neurociencias, Cinvestav Unidad Zacatenco, Ciudad de México, 07360 Mexico

## Abstract

Diego Rivera (1886–1957) painted his first mural *La Creación* (*Creation*, 1922–1923) with an encaustic painting technique, which was traditionally utilized by the ancient Greeks and typically employed beeswax and other resins. Rivera further elaborated that he had innovated a Mexican encaustic technique by incorporating copal, a resin derived from the copal tree. This article presents the novel results of the characterization Diego Rivera’s painting medium by nuclear magnetic resonance spectroscopy, gas chromatography–mass spectrometry, Fourier transform infrared spectroscopy, and optical and scanning electron microscopy of microsamples taken from *Creation* and experimental mock-ups following Rivera’s formula. In the first place, reference standards of the main individual components of beeswax, Mexican copal resin, and elemi resin were analyzed to find the main biomarker molecules. The results obtained confirm the presence of selected biomarkers in the mural samples of copal and elemi resins such as α-amyrin, β-amyrin, and lupeol. Regarding the beeswax compounds, unexpectedly no traces were observed in any of the mural samples. Ethyl phthalate appeared in all mural samples, indicating environmental contamination or the possible application of consolidants with a plasticizer during conservation processes. This article also presents the study of zinc lactate as a degradation product.

## Graphical abstract

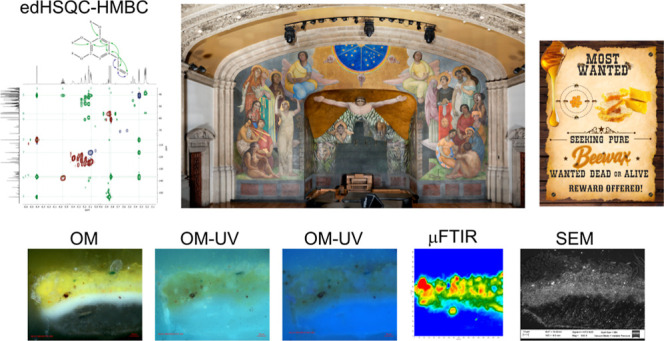

## 1 Introduction

Mexican muralist Diego Rivera painted his first mural, Creation (Figure 1), between 1922 and 1923 at the Amphitheater of the National Preparatory School (today, Antiguo Colegio de San Ildefonso), in Mexico City. In a contemporary interview and many years later, Rivera claimed that he used a wax-based painting technique known as encaustic, with the addition of copal resin to produce a Mexican encaustic recipe.^1,2^ The word copal derives from the nahuatl term copalli (central Mexico indigenous language), which designates a fresh exudate, obtained from trees of genus Bursera. It is a fragrant soluble resin and easy to melt in low temperatures, which comes usually from *B. bipinnata*, *B. vejarvazquezi*, and especially *B. copallifera*, used and extracted since pre-Hispanic times.^3^ The term copal has been applied to two kinds of resins, the freshly collected one and the fossilized one, of the same kind. Nevertheless, in Europe, during the 18th and 19th centuries, the name copal was ascribed to other fossilized resins, hard and reticulated, hard to dissolve, and that is why some treatises discouraged its use, for example, Vibert.^4^ In Mexico, the fresh copal exudate, a fragrant, soluble, and easy to melt in low temperatures, is a common material used as ritual incense since pre-Hispanic times.

**Figure 1:**
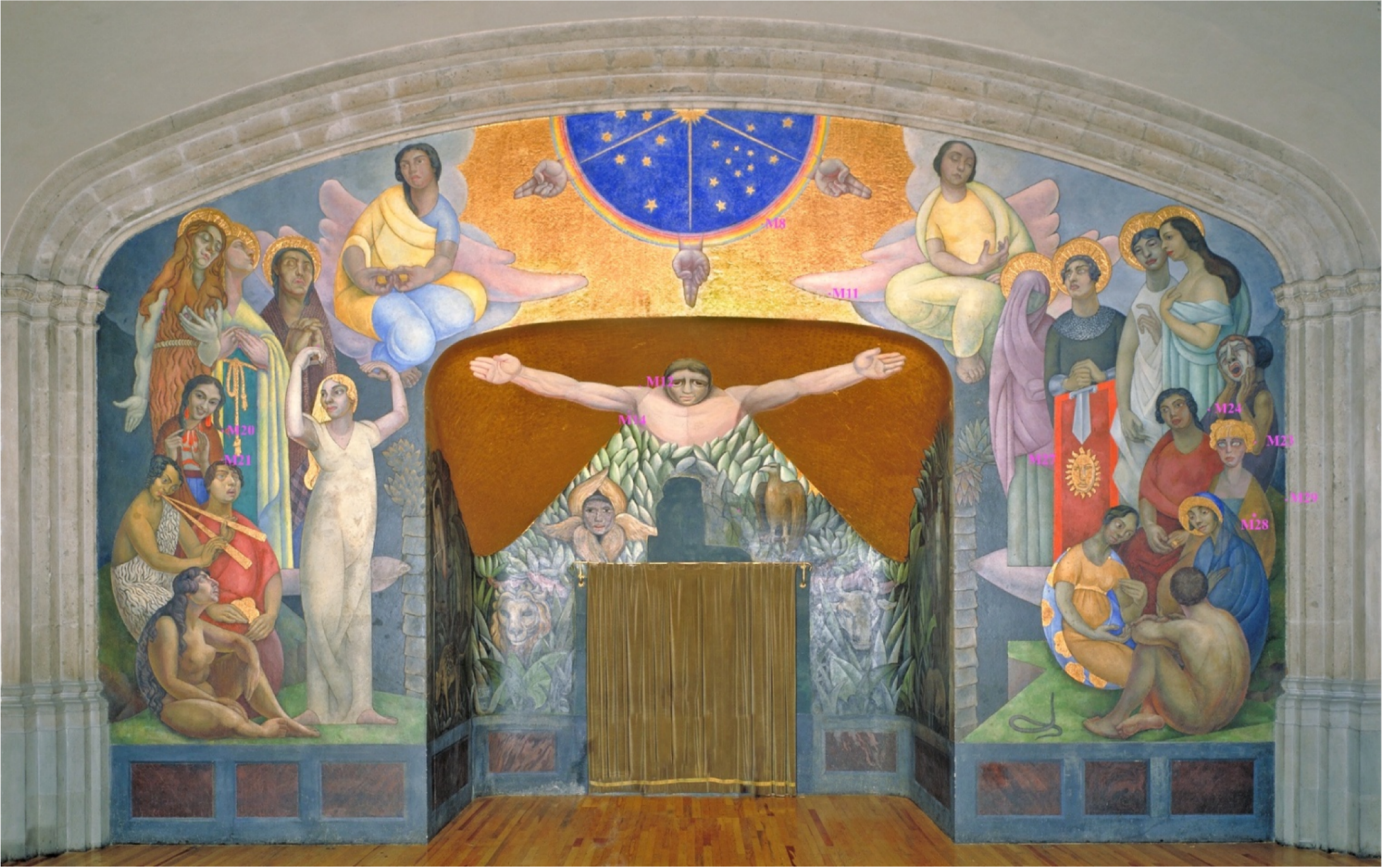
In purple letters is marked the sampling of Creation, Diego Rivera (1922–1923). Photo: Ricardo Alvarado Tapia, 2017, AMFT, Instituto de Investigaciones Estéticas, UNAM. All rights reserved: copyright 2026 *Banco de México, Fiduciario en el Fideicomiso relativo a los Museos Diego Rivera y Frida Kahlo. Av. Five de Mayo No. 2, col. Centro, alc. Cuauhtémoc, c.p. 06000, Ciudad de México. Reproducción autorizada por el Instituto Nacional de Bellas Artes y Literatura (INBAL)*.

During the 1920s, not only Rivera used the encaustic technique but also other younger artists also followed his lead. In Mexico, David Alfaro Siqueiros, Fermín Revueltas, Jean Charlot, and Fernando Leal painted murals with encaustic, in Guadalajara, Jalisco, Carlos Orozco Romero, and in Lima, Perú, José Sabogal.^5^

Rivera declared that he used the ancient Greek painting technique, following the recipes found in the 19th century treatises such as Paillot de Montabert.^6^ Rivera reported his encaustic recipe, which contains a two-part formula: one-half made of one volume of beeswax diluted in a volume of lavender spike oil and the second half of the formula composed of a volume of copal diluted in half a volume of petroleum essence and half a volume of elemi diluted in half a volume of lavender spike oil. Both halves were mixed under a double boiler with the desired pigments. After painting with brushes using the molten mixture, in some areas Rivera cauterized the surface: he applied a blowtorch flame, fueled with gasoline, to give a bright finish.^7^

The formula was adapted by Rivera from de Montabert’s treatise. The copal part of the formula was recommended to paint dark colors, and the wax part better suited the whiter colors; for medium shades, the mixture of both parts was ideal. The fresh copal resin has interesting painting properties because it can be diluted or heated and it is not too sticky, since it has terpene content; but it can also be used as wax or a molding material.

Rivera used lavender spike oil and petroleum essence as solvents, which contain mainly volatile compounds. Lavender spike oil is composed primarily of terpenes, mainly β-linalool (20–43%) and linalool acetate (25–47%).^8,9^ Petroleum essence is a mixture of C_6_–C_12_ aromatic compounds, alkanes, and cycloalkanes.^10,11^ Beeswax is mainly composed of esters and *n*-alkanes, followed by free fatty acids and n-alcohols.^12–14^ Copal and elemi resin (also form the *Burseraceae* family) consists of a complex mixture of organic compounds, mostly of terpenoid origin. Within the triterpenoid fraction, α and β amyrins are commonly reported as major components. Lupeol has also been reported, but its occurrence is restricted to copal and not to elemi. Importantly, the presence of lupeol in copal depends on the specific *Burseraceae* species from which the resin is derived.^15–18^

The characterization of organic materials in cultural heritage, where frequently only very small amounts of material are available, is usually based on separative analytical methods, such as gas chromatography coupled with mass spectrometry (GC–MS) because of its high sensitivity.^19–21^ In addition, high-resolution nuclear magnetic resonance spectroscopy (NMR) provides powerful structural information and has been increasingly applied to the study of organic materials in cultural heritage,^22–26^ including waxes and resins.^27–32^ Complementary, Fourier transform infrared spectroscopy (FTIR) has also been employed in the characterization and degradation study of beeswax and terpene resins.^33–36^

The paper presents the first insights of the material study and chemical characterization of the binding media to understand the history of Diego Rivera’s first mural, its possible degradation processes, and its preservation state. This information may be used to guide the selection of proper conservation and restoration treatments.^37,38^

## 2 Research AIMS

- The aim of the present work is to characterize the binding media from Creation samples using ATR-FTIR, NMR, and GC–MS. First, these analytical methodologies were applied to mock-up samples made following Rivera’s recipe and cauterizing them. After knowing the biomarkers detected in the mock-up samples, we analyzed three original samples taken from the mural.
- Unexpectedly, no beeswax was found in the original samples. So, an experiment to cauterize mock-ups until burning was then conducted to prove if this process could eliminate all detectable traces of beeswax.
- Another objective emerged during the development of the present work was the finding of zinc lactate as a degradation product. This was achieved by analyzing ten selected microsamples taken from the mural with optical microscopy (OM), scanning electron microscopy–energy-dispersive spectrometry (SEM–EDS), and external reflection-Fourier transform infrared microscopy (μER-FTIR).

## 3 Materials and Methods

### 3.1 Reagents

Methanol and diethyl ether were obtained from Fluka Analytical (Steinheim, Germany). Isooctane and *n*-hexane were from Fermont (Monterrey, Mexico). Potassium hydroxide (KOH, ≥85%), hydrochloric acid (HCl, 37%), *N*,*O*-bis­(trimethylsilyl)­trifluoroacetamide (BSTFA), and tridecanoic acid (≥99%) were from Sigma-Aldrich. Deuterated chloroform (CDCl_3_, 99.8% D) was from Cambridge Isotope Laboratories.

For the preparations of mock-ups, local beeswax and copal were bought at Casa Serra artists material shop in Mexico City, ensuring that the same lot was always used; elemi resin was acquired at Kremer Pigments, lavender spike oil was bought at Farmacia Paris, and white spirits was purchased from Windsor & Newton and Mexican Dr. Atl brand.

### 3.2 Mock-Ups

The mock-ups were made following Diego Rivera’s recipe as reported in his treatise on encaustic and fresco, “a mixture made of two dissolutions: (i) one volume of beeswax diluted in a volume of lavender spike oil, and (ii) half a volume of copal diluted in half a volume of petroleum essence and half a volume of elemi diluted in half a volume of lavender spike oil. Both dissolutions were mixed under double boiler”.^7^ The final proportion used were 2 parts beeswax, 3 parts lavender spike oil, 1 part copal, 1 part elemi, and 1 part petroleum. The binder was applied to Portland cement probes (to reproduce the mural original support) and subsequently was cauterized with a propane–butane-fueled blowtorch for 15 s.

### 3.3 Mural Samples

Microsamples (1–2 mg) were taken from Creation after performing imaging and portable spectroscopies analysis (XRF and FORS) (the detailed results of these noninvasive studies will be presented in a forthcoming comprehensive publication). Five microsamples (labeled as M21, M27, M28, M29N, and M29V) were studied by NMR. Three of these (M28, M29N, M29V) were further studied by GC–MS. Nine microsamples (M8, M11, M12, M14, M20, M23, M24, M27, M29) were analyzed by ATR-FTIR. Furthermore, microsamples M8, M11, M14, M20, M24, and M27 were prepared as cross sections for the study of the degradation process using optical microscopy, SEM–EDS, and μER-FTIR.

### 3.4 Nuclear Magnetic Resonance

NMR spectra were acquired using a Bruker Ascend III HD 700 spectrometer (Bruker, Billerica, MA, USA) operating at 16.4 T (700 and 175 MHz for ^1^H and ^13^C frequencies, respectively) and equipped with a 5 mm *z*-axis gradient TCI cryo-probe. The microsamples were dissolved in 0.6 mL of CDCl_3_ and then poured into a 5 mm NMR tube.

Proton (^1^H), carbon (^13^C), homonuclear correlation spectroscopy (COSY), edited heteronuclear single-quantum correlation spectroscopy (ed-HSQC), and gradient heteronuclear multiple-bond correlation spectroscopy (HMBC) experiments were acquired at 300 K using standard Bruker pulse sequences. Data were processed with MestReNova software (v.14.2, Mestrelab Research SL, Santiago de Compostela, Spain). Chemical shifts (δ) in CDCl_3_ are reported in ppm relative to internal TMS.

COSY spectra were acquired with 256 increments of 2K data points, 16 scans, 4 dummy scans, and a recycle delay of 1.3 s ed-HSQC spectra were acquired with 256 increments of 1K data points, 32 scans, 4 dummy scans, and a recycle delay of 2 s. HMBC spectra were obtained with 256 increments of 2K data points, 64 scans, 4 dummy scans, and a recycle delay of 1.4 s. Spectral processing and analysis were made using MestReNova software.

### 3.5 Gas Chromatography/Mass Spectrometry

Samples were analyzed using an Agilent 7890B gas chromatography system coupled to an Agilent 5977A mass spectrometer (Agilent Technologies, Santa Clara, CA, USA). Separation was achieved with an HP-5MS column (30 m × 0.25 mm × 0.25 μm). The oven program was set as follows: initial temperature at 80 °C, held for 2 min, raised at 10 °C/min up to 200 °C, raised again at 6 °C/min up to 280 °C, where it was held for 15 min. Injection was carried out in split mode (20:1) at 280 °C. Carrier gas: helium (purity 99.9995%) was used as the carrier at a constant flow of 1.2 mL min^–1^. Transfer line and ion source temperatures were 300 and 230 °C, respectively. The electroionization potential was set at 70 eV. A quadrupole mass analyzer was used in SCAN mode, acquiring the spectra from *m*/*z* 50 to 500. Data were processed using MassHunter Workstation Software (Unknown Analysis and Qualitative Analysis modules, v. B.07.00; Agilent Technologies, Santa Clara, CA, USA). Compound identification was performed by comparison with the NIST 14 mass spectral library.

The analytical procedure used was based on that reported previously,^39^ with slight modifications in the derivatization process. A sample (1–3 mg) was subjected to alkaline hydrolysis by adding 1 mL of methanolic KOH [KOH–CH_3_OH (10% weight)/KOH–H_2_O (10% weight), 2:3] and heating at 60 °C for 3 h. After hydrolysis, neutral organic components were extracted with *n*-hexane (500 μL × 3). After acidification with hydrochloric acid (10 M; to pH 2), the acidic organic components were extracted from the hydrolysate with diethyl ether (500 μL × 3). Aliquots of both fractions were evaporated to dryness under a gentle stream of nitrogen and reconstituted in 150 μL of iso-octane. The acidic fraction was then mixed with an internal standard solution (10 μL *n*-tridecanoic acid, 1.52 μg mL^–1^) and derivatized with 25 μL of BSTFA at 80 °C for 30 min. The neutral fraction was analyzed directly, without the addition of internal standard or derivatization, since partial derivatization of its triterpenoids had been observed in preliminary analyses when derivatization was attempted (Figure S1). 1 μL of each solution was injected for GC–MS analysis; however, for the acidic fraction, when no signals were observed, 3 μL was injected to ensure that no traces were present.

### 3.6 ATR-FTIR

ATR-FTIR analysis was carried out directly on the samples without any prior preparation using a Cary 600 FTIR spectrophotometer (Agilent Technologies, Santa Clara, CA, USA). Spectra were recorded in the 4000–400 cm^–1^ range, with a resolution of 4 cm^–1^ and by averaging 128 scans. Data processing was performed with OriginPro (2024, Northampton, Massachusetts, EE. UU.) and Spectragryph software (v.1.2, Oberstdorf, Germany). All experimental ATR-FTIR spectra in the manuscript are reported as raw data. Band assignments were made based on published references (Table S1).

### 3.7 Optical Microscopy

The microsamples were prepared as cross sections; first, they were embedded in methacrylic resin ClaroCit (Roper Technologies, Sarasota, FL, USA). Then, they were polished with SiC abrasive papers ranging from 800 to 4000 grit. The prepared cross sections were examined under an Axio Imager Z2 optical microscope (Carl Zeiss, Oberkochen, Germany), equipped with a Xenon arc lamp for UV fluorescence, employing filters of 430–465 nm and 465–500 nm, as well as a HAL100 light source for reflected light imaging, using polarization, dark- and bright-field filters.

### 3.8 SEM–EDS

The study of the micromorphology and composition of samples prepared as cross sections was conducted using an EVOMA25 scanning electron microscope (Carl Zeiss, Oberkochen, Germany), equipped with a 30 mm EDS microprobe (Bruker, Bremen, Germany). Microsamples were mounted on a double-sided carbon tape to ensure conductivity. Backscattered electron (BSE) images were obtained under variable pressure conditions (80 Pa) with a nitrogen flow. EDS spectra and elemental distribution maps were collected at an accelerating voltage of 15.0–20.0 kV.

### 3.9 μER-FTIR

Chemical maps were acquired with an Agilent Cary 620 FTIR microscope equipped with a 64 × 64 focal plane array (FPA) detector that was coupled to an Agilent Cary 670 FTIR spectrometer (Agilent Technologies, Santa Clara, CA, USA). The data were acquired with a spatial resolution of 1.1 μm per pixel and subsequently processed with Agilent Resolutions Pro software. The field of view (FOV) was 70 × 70 μm. Spectra were collected in the 4000–900 cm^–1^ range, with an average of 128 scans at a resolution of 4 cm^–1^.

## 4 Results and Discussion

### 4.1 Analysis of Rivera’s Encaustic Mock-Up by ATR-FTIR

The raw materials in Rivera’s formulation were analyzed by ATR-FTIR prior to binder incorporation to establish infrared reference spectra and to detect changes after cauterization. The analyzed materials included copal, elemi, beeswax, lavender spike oil, white spirit, and zinc white. The characteristic band assignments are summarized in Table S1. As shown in Figure 2, the spectrum acquired after cauterization revealed a decreased intensity of the elemi band at 1589 cm^–1^, an attenuation of a band at 1540 cm^–1^ (not attributable to any of the raw materials), and a reduced intensity of the lavender spike oil bands at 1415, 1216, 919, 841, and 691 cm^–1^.

**Figure 2:**
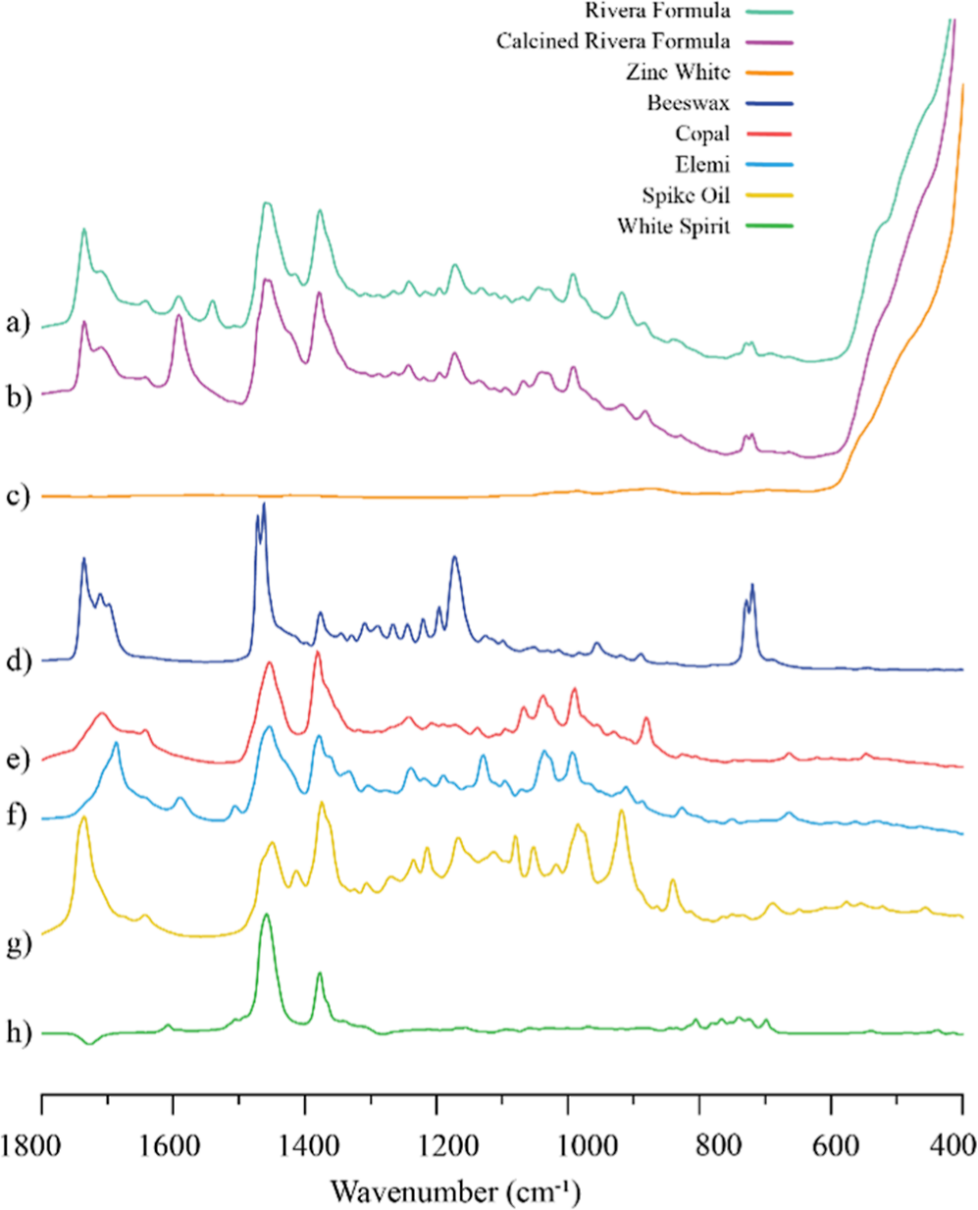
ATR-FTIR spectra of reproduction of Rivera’s encaustic formula and reference materials: (a) uncauterized mock-up, (b) cauterized mock-up, (c) zinc white pigment, (d) beeswax, (e) copal (f) elemi, (g) lavender spike oil, and (h) white spirit.

### 4.2 Analysis of Rivera’s Encaustic Mock-Up by NMR

The ^1^H NMR spectra of the mock-up sample in CDCl_3_ allowed the identification of the major components in the mixture. This was achieved by analyzing the spectral data and comparing it both with the spectra of the raw materials and with published ref 28. Table S2 summarizes the chemical shifts of the main resonances observed. Some characteristic and isolated signals in the ^1^H NMR spectra include for beeswax, esters (4.90, 4.80, 4.05, 2.28 ppm), alcohols (3.79, 3.64, 3.53, ppm), and fatty acids (5.40, 2.34, and 2.01 ppm); for copal and elemi, α-amyrin (5.12 ppm), β-amyrin (5.18 ppm), and lupeol (4.68, 4.56, 3.22, and 1.67 ppm); for elemi, elemicin (6.41, 3.85, and 3.82 ppm); for copal an unknown compound (3.38 ppm); and for lavender spike oil, linalool (5.91, 5.21, 5.06, 2.00, ppm). The chemical structures of these markers were elucidated using HSQC-ed, HMBC, and COSY spectra.

Figure 3 shows the ^1^H NMR spectra of Rivera’s painting replica before and after cauterization, along with the proton spectra of the raw materials. Molecular markers were identified for each raw material, and their assignments as well as the ^1^H and ^13^C chemical shifts are provided in Figures S2–S13 and Tables S3–S14. In the case of White Spirit, no characteristic signals were detected, owing to the volatile nature of its constituent compounds.

**Figure 3:**
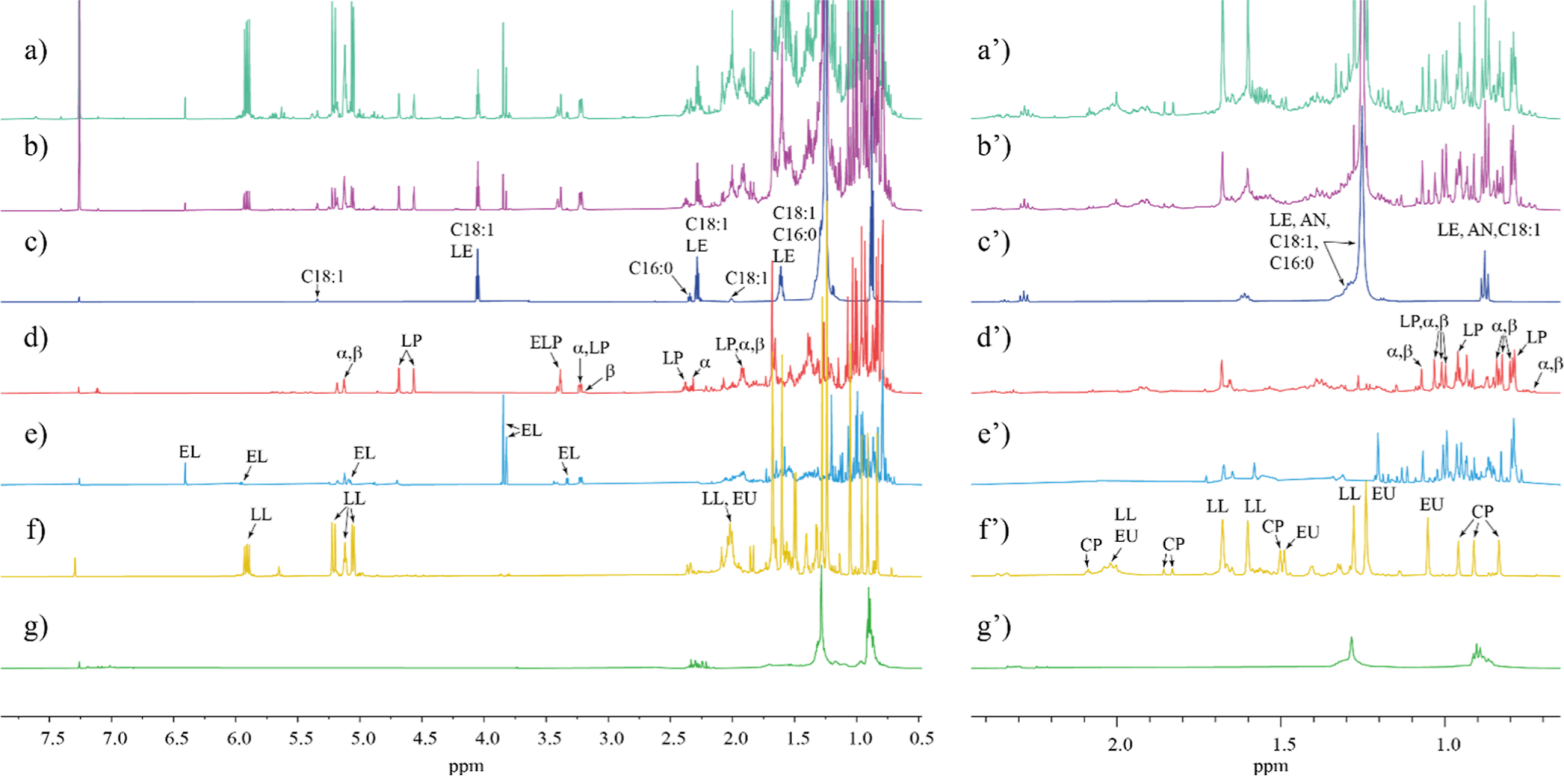
^1^H NMR spectra (700 MHz, 300 K, CDCl_3_) from 7.9 to 0.5 ppm of the reproduction of Rivera’s encaustic formula: (a) uncauterized mock-up, (b) cauterized mock-up, (c) beeswax, (d) copal, (e) elemi, (f) lavender spike oil, and (g) white spirit. (a′–g′) Expanded ^1^H NMR spectra of raw materials from 2.4 to 0.6 ppm. Chemical markers elucidated by ed-HSQC, HMBC, and COSY (Figures S2–S13) are indicated: C18:1 (oleic acid), C16:0 (palmitic acid), LE (linear esters), AN (long-chain alkanes), α (α-amyrin), β (β-amyrin), LP (lupeol), EPL (epilupeol); EL (elemi), LL (linalool), CP (camphor), and EU (eucalyptol).

The characteristic proton signals of the molecular markers shown in Figure 3 are described as follows:

- Beeswax: oleic component at 0.88, 1.24–1.31, 1.61, 2.28, and 5.35 ppm. Palmitic acid at 0.88, 1.64, 2.34, and 1.24–1.31 ppm. Linear esters at 0.88, 1.24–1.31, 1.61, 2.28, and 4.06 ppm. Linear alkanes at 0.88 and 1.24–1.31 ppm.
- Elemi resin: elemicin at 3.33, 3.84, 5.08, 5.96, and 6.40 ppm.
- Lavender oil: linalool at 1.28, 1.60, 1.68, 2.01, 5.05, 5.21, and 5.91 ppm. Limonene at 1.91, 2.38, 4.57, 4.69, and 5.13. Camphor was at 0.84, 0.91, 0.96, 1.50, 1.83, 2.09, and 2.33 ppm. Eucalyptol at 1.05, 1.24, 1.41, 1.50, 1.65, and 2.01 ppm.
- Copal resin: Lupeol at 0.77, 0.79, 0.99, 1.03, 2.37, 3.22, 4.59, and 4.69 ppm. Epilupeol 3.38 ppm. α-amyrin at 0.82, 0.84, 1.00, 1.01, 1.07, 1.90, 2.07, 3.22, and 5.12 ppm. β-amyrin at 0.84, 1.00, 1.01, 1.13, 3.18, and 5.12 ppm.

The ^1^H NMR spectra of Rivera’s encaustic replica after cauterization shows a general decrease in the characteristic signals corresponding to the molecular markers of the raw materials. Both spectra, before and after cauterization, were normalized using the lupeol signals at 4.59 and 4.69 ppm.

### 4.3 Analysis of Rivera’s Encaustic Mock-Up by GC–MS

Similar to the NMR results, several molecular markers from the materials used in Rivera’s encaustic mock-up were identified. In the neutral fraction (Figure 4a, Table S15), a series of long-chain alkanes (predominantly odd-numbered homologues such as A27, A29, and A31) attributable to beeswax were detected, together with triterpenoids such as α- and β-amyrin and lupeol, characteristic of copal and elemi. In addition, elemicin and isoelemicin, more specific markers of elemi, were observed. It should be noted, however, that the presence of both odd- and even-numbered alkanes suggests that the beeswax employed may not correspond to a pure material but rather to a commercial formulation potentially containing a minor proportion of paraffin or other petroleum-derived waxes. Although the wax markers do not entirely coincide with those reported,^39^ the essential point is the demonstration that no wax was detected in the paint samples. The acidic fraction (Figure 4b, Table S16) contained palmitic and stearic acids, consistent with beeswax-derived components, whereas volatile organic compounds expected from copal, elemi, and lavender spike oil were not detected, suggesting substantial loss during mock-up preparation. Several terpenoids from copal and elemi were also absent, consistent with volatilization during mixture preparation.^13^

**Figure 4:**
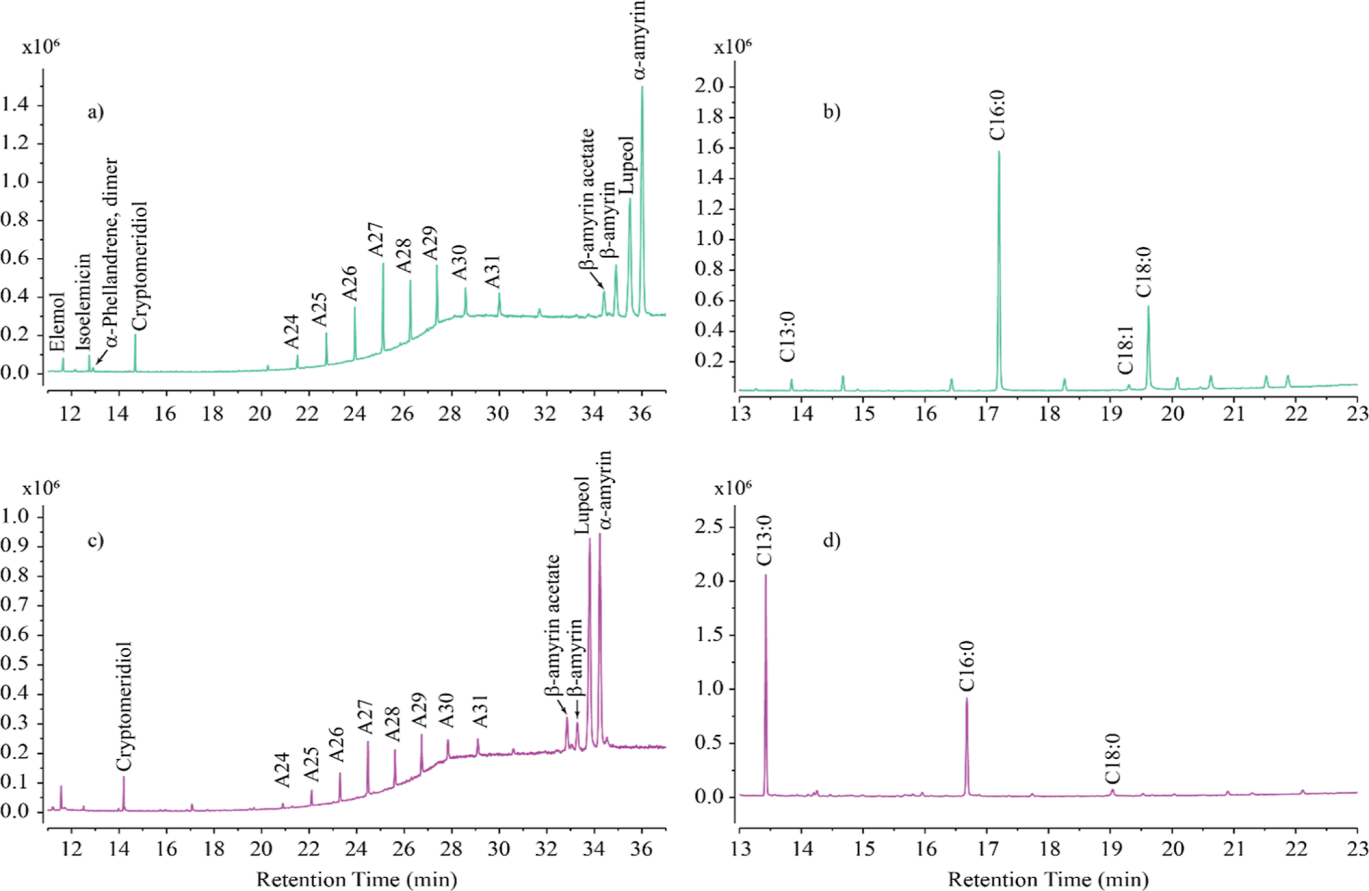
Total ion chromatograms of Rivera formula reproduction: (a,b) uncauterized mock-up and (c,d) cauterized mock-up; (a,c) neutral and (b,d) acid fractions. Labeled compounds: A (alkanes), C16:0 (palmitic acid), C18:0 (stearic acid) and C18:1 (oleic acid), C13:0 (tridecanoic acid, internal standard). Note: retention times differ between the chromatograms due to a change of column during the analyses.

After cauterization, the main copal markers in the neutral fraction (α-amyrin, β-amyrin, β-amyrin acetate, and lupeol) were still detected, indicating thermal stability under the applied treatment conditions (Figure 4c). In comparison with the noncauterized samples, cauterization led to a change in the relative α-amyrin/lupeol signal relationship, resulting in peaks of comparable height. A relative decrease of beeswax-related alkane peaks was also observed.

#### 4.3.1 Analysis of Creation Mural Samples by ATR-FTIR

Figure 5 presents the ATR-FTIR spectra of samples from Creation (M8, M11, M12, M14, M20, M23, M24, M27 and M27). These spectra were compared to those of the raw materials as well as with reference spectra of inorganic fillers from the RRUFF database. Through this comparison, the presence of copal, calcite, barite, talc, and gypsum was identified (Figures S14–S22).

**Figure 5:**
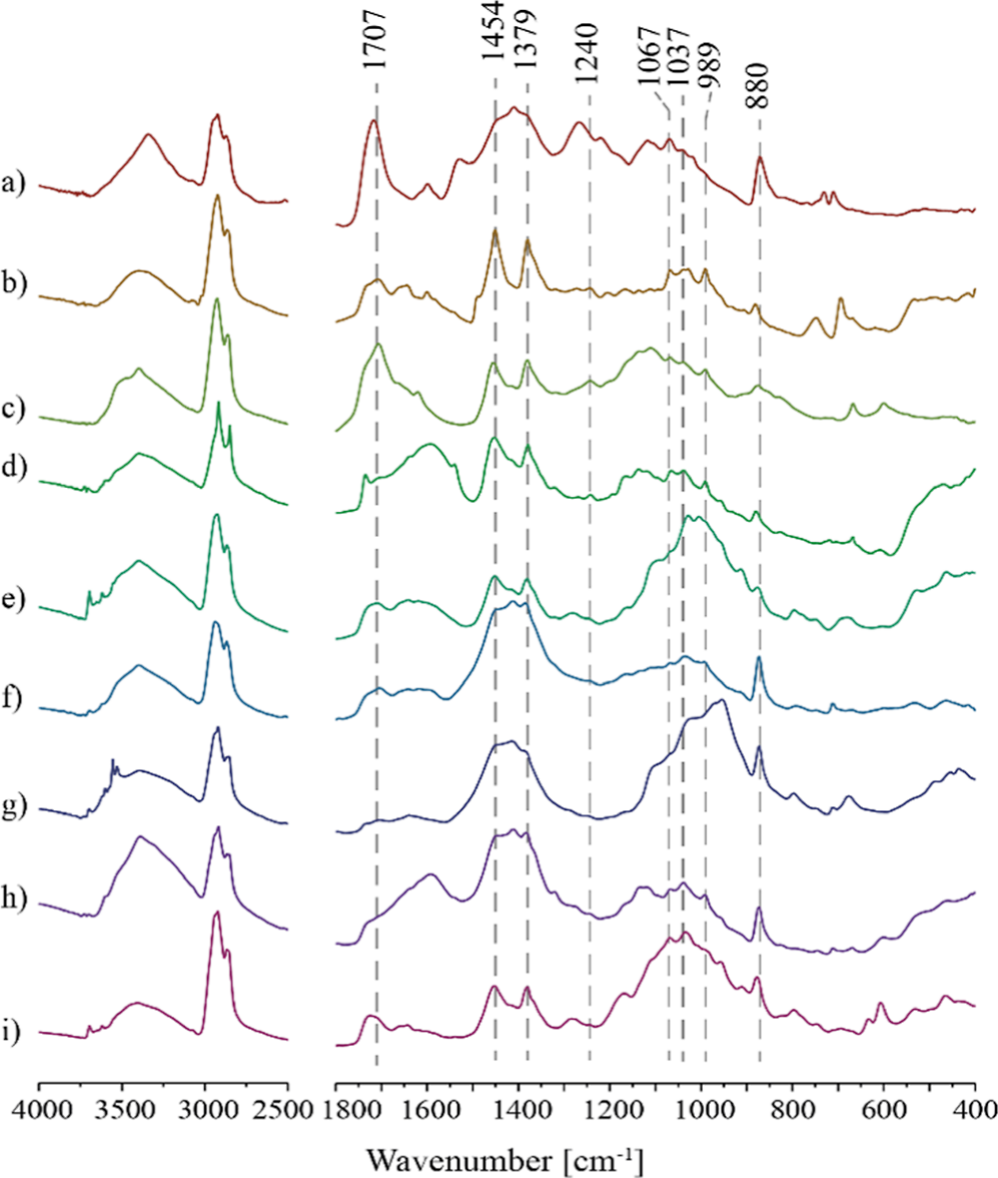
ATR-FTIR spectra of Creation samples: (a) M8, (b) M11, (c) M12, (d) M14, (e) M20, (f) M23, (g) M24, (h) M27, and (i) M29. Characteristic bands of copal are indicated.

The identification of the main compounds was supported by the assignment of characteristic FTIR bands for each material. Copal was confirmed by its signals at 1707 cm^–1^ (C=O stretching), 1454 cm^–1^ [δ­(CH_2_)], 1379 cm^–1^ [δ­(CH_3_)], 1244 cm^–1^ [δ­(C–H)], 1067 cm^–1^ [ν­(C–O–C)], 1037 cm^–1^ [ν­(C–O)], 989 cm^–1^ (C–O), and 880 cm^–1^ (exocyclic methylene groups).^40^ Calcite was identified through its bands at 1414 cm^–1^ [ν­(CO_3_^2–^)], 871 cm^–1^ [δ­(O–C–O)], and 711 cm^–1^.^41^ Gypsum exhibited broad O–H stretching bands around 3400 cm^–1^, together with signals at 1620 cm^–1^, 1110 cm^–1^ (asymmetric SO_4_^2–^ stretching), 667 cm^–1^, and 600 cm^–1^.^41^ Talc showed absorption bands at 3673, 1000, and 667 cm^–1^, whereas barite was characterized by its asymmetric SO_4_^2–^ stretching at 1169 cm^–1^ and additional bands at 1067, 981, 631, and 605 cm^–1^.^41^

In contrast to the mock-ups, no characteristic beeswax signals were observed, such as the bands 729 and 719 cm^–1^ [rocking (CH_2_)]. These bands correspond to a spectral region where none of the other raw materials exhibit intense absorption, making the absence of these beeswax markers particularly notable.

### 4.4 Analysis of Creation Mural Samples by NMR

Figure 6 shows the ^1^H NMR spectra of microsamples of the mural M21, M27, M28, M29N, and M29V, along with the spectra of Rivera’s cauterized painting reproduction highlighting the identified chemical markers. A signal at 3.20 ppm was assigned to lupeol and α-amyrin, while the signal at 3.38 ppm was assigned to epilupeol; these three compounds are characteristic components of copal resin. Additional signals at 4.69 and 4.59 ppm could also correspond to limonene, since both limonene and lupeol share an isopropylene substituent. The structure of limonene was elucidated and confirmed in sample M29N (Figure S23, Table S17). Furthermore, a broad signal at 3.8 ppm was detected; although its precise assignment remains uncertain, it is likely related to copal as well.

**Figure 6:**
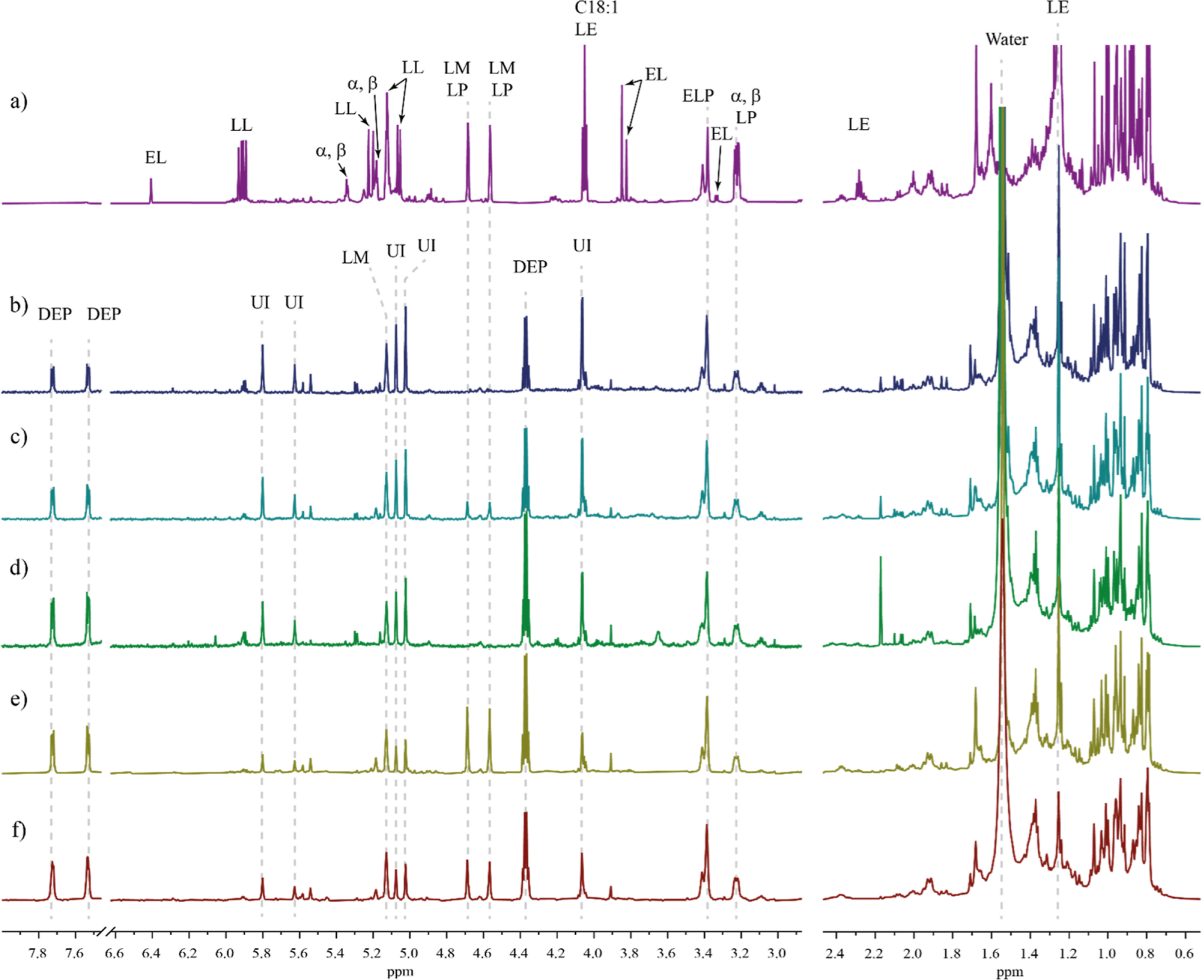
^1^H NMR spectra (700 MHz, 300 K, CDCl_3_) of (a) cauterized mock-up and (b–f) Creation samples: (b) M21, (c) M27, (d) M28, (e) M29N, and (f) M29V. The identified chemical markers are indicated: LE (linear esters), α (α-amyrin), β (β-amyrin), LP (lupeol), EPL (epilupeol); EL (elemi), LL (linalool), LM (limonene), DEP (diethyl phthalate). Unidentified signals are labeled as UI.

The broad signal at 1.24 ppm could be attributed to the long-chain methylene groups. However, the characteristic triplet at 0.88 ppm, which is typical of linear alkanes, was not observed in the mural samples. Instead, a triplet at 2.29 ppm was detected, which could correspond to a fatty acid such as palmitic acid or to linear esters. Therefore, the presence of long-chain linear alkanes, and consequently beeswax, cannot be confirmed by NMR based solely on the 1.24 ppm signal.

Signals at 5.79, 5.62, 5.07, 5.02, and 4.06 ppm were observed in the spectra of the mural sample but none could be conclusively identified. The signal at 4.06 ppm overlaps with the methylene triplet bound to the ester oxygen; nevertheless, its multiplicity and HMBC correlations (data not shown) differ from those expected for such a group, discarding the presence of linear ester and, consequently, beeswax.

The mural samples also show intense signals of ethyl phthalate, with two doublets of doublets at 7.51 and 7.71 ppm and a quadruplet in 4.37 ppm (Figure S24, Table S18).

Finally, no signals corresponding to elemicin or linalool were detected in any of the mural microsamples. Thus, the presence of elemi resin or lavender oil could not be confirmed.

#### 4.4.1 Analysis of the Creation Mural Samples by GC–MS

Figure 7 shows the total ion chromatograms of both the mural microsamples and the encaustic mock-up reference. In the total ion chromatograms of three samples from Creation (Figure 7b–d), the presence of β-amyrin acetate, lupeol, and α-amyrin confirms the use of a triterpenoid resin consistent with copal, in agreement with the NMR and FTIR results. The α-amyrin peak showed lower intensity than that of lupeol, similar to the relative signal change observed in the cauterized mock-up (Figure 7a). The α-phellandrene dimer, present in both copal and elemi, was also observed. Additionally, signals attributable to the solvents used in the mixture were also detected, including 2-bornanone, endoborneol, linalool, and linalool oxide. In contrast, long-chain alkanes associated with beeswax were not detected in the mural microsamples, and no beeswax-related compounds were observed in any of the analyzed samples.

**Figure 7:**
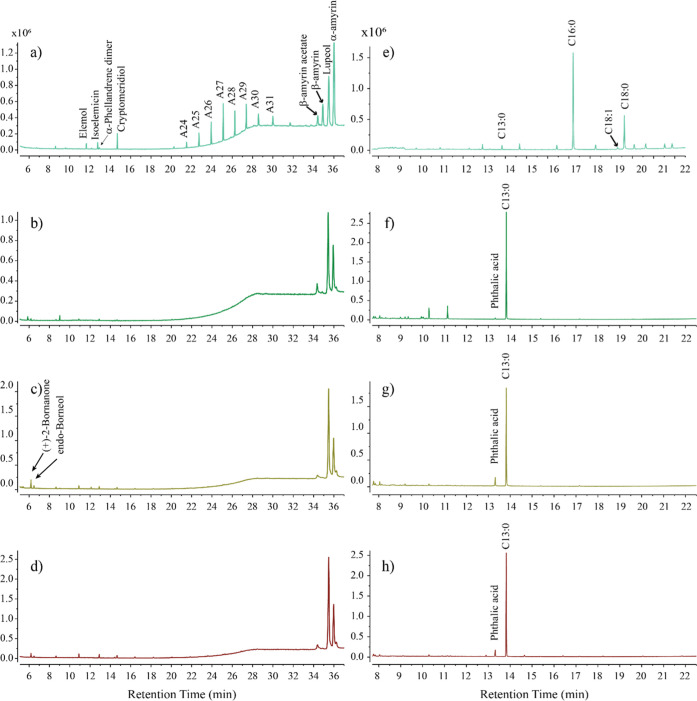
Total ion chromatograms of (a–d) neutral and (e–h) acid fraction (a,e) of encaustic mock-ups and Creation mural samples: (b,f) M28, (c,g) M29N, and (d,h) M29V. Labeled compounds: A (alkanes); C16:0 (palmitic acid), C18:0 (stearic acid) and C18:1 (oleic acid), C13:0 (tridecanoic acid, internal standard).

In the acid fraction of the three mural samples, phthalic acid and palmitic acid were identified, although the latter was present only at a noise-level intensity (Figure 7f–h). In contrast, phthalic acid was not observed in the acid fraction of the mock-up (Figure 4e).

#### 4.4.2 Degradation Study by μER-FTIR

Figure 8 OM presents the chemical maps of samples M8, M11, M14, M20, M24, and M27, showing the distribution of the band at 1320 cm^–1^ accompanied by polarized light micrographs to provide a high-resolution reference for the analyzed regions. For each map, the μER-FTIR spectra were extracted from the areas with the highest signal intensity at 1320 cm^–1^ (indicated by red regions on each map). The extracted and processed μER-FTIR spectra were then compared with ATR-FTIR reference spectra of commercial zinc lactate. The presence of Zn in the cross sections of the samples analyzed by μER-FTIR was confirmed by SEM–EDS (Figures S24–S29 and Tables S19–S24). In addition to the band at 1320 cm^–1^, the extracted spectra display characteristic signals at 1600 (ν_as_ COO^–^), 1480, 1406, 1273, 1118­(ν_s_ C–O), 1092 (ν_s_ C–O), and 1047 cm^–1^.^42^ Signals at 1364 and 1320 cm^–1^ can also be attributed to metallic oxalates such as calcium or zinc, which are also common degradation products. Combined with the detection of Zn by SEM–EDS, these spectral features confirm the presence of zinc lactate within the cross sections of the samples. Table S25 shows a comparison of the chemical maps with optical and electron microscopy of the samples where zinc lactate was identified.

**Figure 8:**
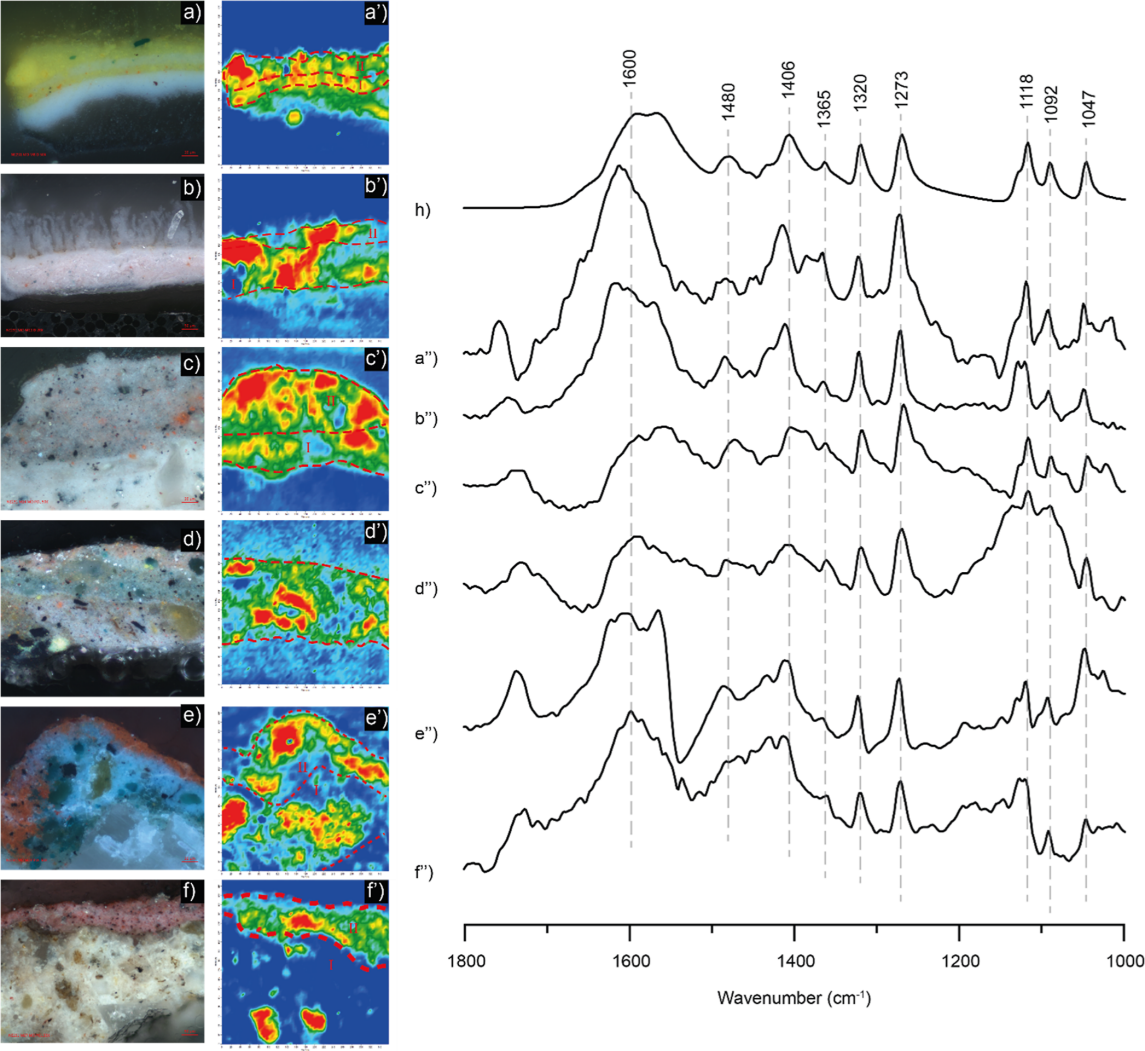
Polarized light OM micrographs (a–f) and corresponding μER-FTIR (a′–f′) analysis of cross-sectional samples: M8, M11, M14, M20, M24, and M27. μER-FTIR spectra (a″–f″) extracted from each map, with bands attributed to zinc lactate and zinc oxalate indicated. (h) ATR-FTIR reference spectrum of zinc lactate.

This study focuses only on the pigments relevant for the degradation processes; the complete Diego Rivera painting palette will be published later.

## 5 Discussion

In all of the results obtained from all of the techniques used in this study, only copal resin markers were clearly identified in the mural samples. The precise compositions of the solvents could not be determined. However, NMR analysis revealed a higher proportion of limonene relative to linalool, suggesting the use of a solvent different from spike oil or possibly a mixture of several solvents. Rivera also mentioned the use of petroleum as a solvent, but its highly volatile composition would leave no markers in the sample.

As previously mentioned, Diego Rivera and his assistants described the use of a blow torch and the cauterization of the mural surface. Therefore, one hypothesis about the absence of beeswax markers was that cauterization may have caused the sublimation of beeswax.

The sublimation of wax due to the decay process has been reported; however, some characteristic markers of beeswax and degradation products can still be detected even in highly aged, multicomponent historical artifacts.^33,35^ The binder may suffer the sublimation of some volatile compounds in a dry and warm environment, but the complete disappearance of chemical markers has never been reported.^35,43^

In the mock-up samples after cauterization, up to the point of calcination, NMR and ATR analyses showed reduced signal intensities but the beeswax markers never disappeared entirely. Before and after the cauterization, NMR consistently detected beeswax compounds like esters, alcohols, and fatty acids. Also, copal and elemi compounds like α- amyrin, β-amyrin, lupeol, and elemicin were always detected. Linalool, a spike oil compound, was also found. GC–MS analysis of the same mock-up samples revealed long-chain alkanes (C_25_–C_31_) of beeswax, β-amyrin, α-amyrin, lupeol, and elemicin in the neutral fraction, and palmitic and stearic acids in the acid fraction.

Infrared spectroscopy of the mural samples revealed the presence of inorganic fillers, such as calcite, gypsum, talc, and barite. Copal was identified as the binder in all of the samples analyzed. Its characteristic bands at 1454 and 1379 cm^–1^, attributed to CH_2_ and CH_3_ bending vibrations, were consistently detected. Although these signals are also present in the spectrum of elemi, other diagnostic elemi markers, such as the CH_2_ bending at 1589 cm^–1^ or the band at 1507 cm^–1^, were absent. Beeswax was not detected by this technique in any of the samples.

Based on these results, the hypothesis changed to propose a different composition of Diego Rivera’s formula for Creation mural, which only contained copal as a binder.

The ^1^H NMR spectra of the mural samples showed intense signals of ethyl phthalate in all of the analyzed samples. Given the relatively high signal intensity of the signal of ethyl phthalate, the possibility of contamination of the surface by atmospheric pollution is not the most possible explanation, although it cannot be discarded because the mural is in downtown Mexico City, a heavily polluted area. Phthalates have been reported in indoor surfaces.^44,45^ Also, in the acid fraction by GC–MS, phthalic acid was identified. An alternative explanation is that ethyl phthalate resulted from conservation processes, as this compound is a common plasticizer found in almost all plastic containers used for solvents.^46,47^

The μER-FTIR analysis of the mural samples identified zinc lactate as a degradation product of the mixture of zinc oxide and copal resin. The presence of short-chain zinc carboxylates in terpene resins has been reported, although no degradation mechanism has been proposed so far, likely due to the complex composition of these resins.^48–51^

The formation of zinc lactate could be associated with the cauterization of the terpene-based binder. Isoprene has been previously reported as a combustion product generated during the thermal degradation of monoterpenes and diterpenes.^52–54^ Among isoprene oxidation products, methylglyoxal is a known intermediate in the ZnO-catalyzed synthesis of lactic acid from glucose or glycerol.^55–57^ Accordingly, the presence of lactic acid in the mural can be attributed to the generation of isoprene oxidation products during the mural’s manufacture, followed by their catalytic conversion to lactic acid and subsequent coordination with Zn^2+^ ions, leading to zinc lactate formation. Zinc lactate has also been identified in oil paints.^42,58–60^ In a previous work, we proposed a radical-catalyzed pathway for its formation in oil media; nevertheless, further studies are required to elucidate the specific degradation mechanism in terpene resins.^61^

## 6 Conclusion

The analytical results obtained in this study do not correspond to the recipe that Diego Rivera described when he was painting in 1923 or at a later time. The most surprising fact was that no chemical markers from beeswax were detected in the mural samples.

The results suggest that the encaustic medium prepared by Rivera contained mainly copal resin, probably diluted with spike/lavender oil, and/or petroleum.

Many other encaustic recipes from contemporary artists, as José Sabogal, Carlos Orozco Romero, and Fermín Revueltas, that followed the Diego Rivera encaustic technique include beeswax along with copal. In our lab, the characterization of samples of such murals confirmed the presence of beeswax, but this work is still in process and will be published later. Although it was surprising not to find wax, it is known from historical practice that copal can be used as a painting medium, and it has proven to be quite effective as such. Even if there is no wax in the composition of the mural, it still may be considered an encaustic technique since it has been cauterized. The name of the technique makes a reference to the cauterization process, and in fact there is no single recipe for encaustic or a material that gives the name to it.

## Supplementary Material



## Notes

Figures S1: TIC of the derivatized uncauterized mock-up; S2–S13: ed-HSQC and HMBC spectra of standards; Figures S14–S22: ATR-FTIR spectra of microsamples from Creation; Figures S23–S24: ed-HSQC and HMBC spectra from Creation; Figures S25–S30: SEM–EDS micrographs of samples from Creation; Table S1: ATR-FTIR characteristic absorptions of commercial binders; Tables S2–S14: NMR data of reference sample; Tables S15–S16: compounds identified by GC–MS in mock-up and mural samples; Tables S17–S18: NMR data of sample from Creation; Tables S19–S24: mass percentages of elements identified by SEM–EDS in samples from Creation; and Table S25: microscopy analysis of cross-sectional sample from Creation (PDF)

## ⊥

Instituto de Investigaciones Estéticas, Universidad Nacional Autónoma de México, ciudad de México
