# Supplementary material for: An Encaustic without Wax: Microanalytical Binder Characterization of Diego Rivera’s First Mural, Experimental Reproduction, and Identification of Degradation Products
Source: ACS Omega. 2026 Jul 15;11(29):43265–76. doi: 10.1021/acsomega.6c00983 (PMC13425480; doi:10.1021/acsomega.6c00983)
Supplement: Supplementary file 1 [file ao6c00983_si_001.pdf]

An encaustic without wax: Microanalytical binder characterization of Diego Rivera's first mural, experimental reproduction, and identification of degradation products

Pablo Aguilar-Rodríguez [a,§,d], Joel Landa-Huerta [a,§], Angel Santiago-Jiménez [a,§], Sandra Zetina [b,§], Araceli Peña-Álvarez [c]; Mayra León-Santiago [a,§], Nuria Esturau-Escofet [*a,§].

[a]Instituto de Química, Universidad Nacional Autónoma de México, ciudad de México.

[b]Instituto de Investigaciones Estéticas, Universidad Nacional Autónoma de México, ciudad de México.

[c]Facultad de Química, Universidad Nacional Autónoma de México, ciudad de México.

[d] Currently at Instituto de Investigaciones Estéticas, Universidad Nacional Autónoma de México, ciudad de México.

[§]Laboratorio Nacional de Ciencias para la Investigación y la Conservación del Patrimonio Cultural (LANCIC, National Science Laboratory for Research and Conservation of Cultural Heritage)

[illegible] 1
[illegible] 3
[illegible] 4
[illegible] 5
[illegible] 6
[illegible] 7
[illegible] 8
[illegible] 9
[illegible] 10
[illegible] 11
[illegible] 13
[illegible] α-amyrin with key correlation and the assignment of the signals in the spectra are shown. 15
[illegible] β-amyrin with key correlation and the assignment of the signals in the spectra are shown. 17
[illegible] 20
[illegible] 20
[illegible] 21
[illegible] 21
[illegible] 22
[illegible] 22
[illegible] 23
[illegible] 23
[illegible] 24
[illegible] 25
[illegible] 26
[illegible] 27
[illegible] 28

[illegible] ... 29
[illegible] ... 30
[illegible] ... 31
[illegible] ... 32

[illegible] ... 1
[illegible] ... 2
[illegible] δ_H, δ_C [illegible] ... 3
[illegible] δ_H, δ_C [illegible] ... 4
[illegible] δ_H, δ_C [illegible] ... 5
[illegible] δ_H, δ_C [illegible] ... 6
[illegible] δ_H, δ_C [illegible] ... 7
[illegible] δ_H, δ_C [illegible] ... 8
[illegible] δ_H, δ_C [illegible] ... 9
[illegible] δ_H, δ_C [illegible] ... 10
[illegible] δ_H, δ_C [illegible] ... 12
[illegible] δ_H, δ_C [illegible] ... 14
[illegible] δ_H, δ_C [illegible] α-amyrin identified in copal from Casa Serra. ... 16
[illegible] δ_H, δ_C [illegible] β-amyrin identified in copal from Casa Serra ... 18
[illegible] ... 19
[illegible] ... 19
[illegible] δ_H, δ_C [illegible] ... 25
[illegible] δ_H, δ_C [illegible] ... 26
[illegible] ... 27
[illegible] ... 28
[illegible] ... 29
[illegible] ... 30
[illegible] ... 31
[illegible] ... 32
[illegible] ... 33

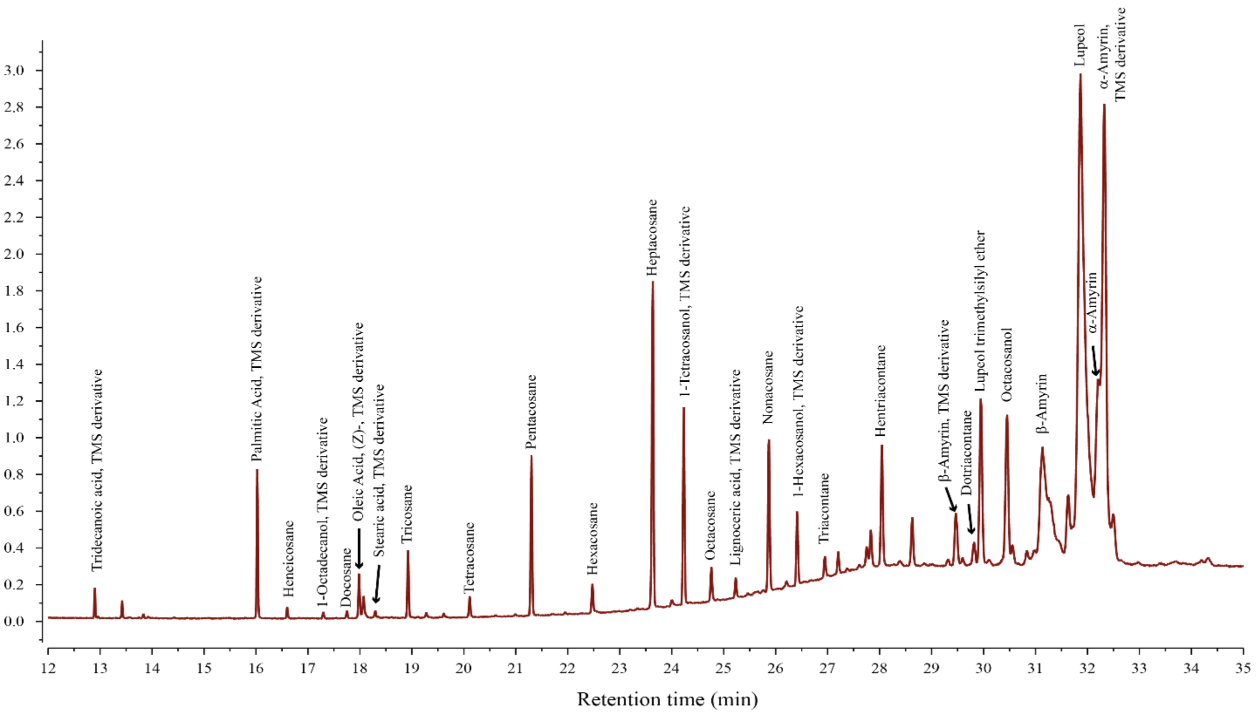

[illegible]

[illegible]

Sample	Wave number [cm^{-1}]	Reference
Copal	1707 ν(C=O), 1454 $\delta(CH_2)$, $\delta(CH_3)$, 1379 $\delta(CH_3)$, 1244 δ(C-H), ν(C-O-H) 1138 ν_s(C-O-C), 1093 O-H, ν_s (C-O-C), 1067 ν_s(C-O-C), 1037 ν_s(C-O), 989 C-O, 957, 880 Exocyclic methylene groups, 546 Unsaturated bonds cm^{-1}	(Martín-Ramos et al., 2018)
Beeswax	2955 $\nu_{as}(CH_3)$, 2916 $\nu_{as}(CH_2)$, 2848 $\nu_s(CH_2)$, 1735 ν(C=O), 1471 δ deformation (CH_2), 1462 δ deformation (CH_2), 1376, 1345 $\omega + \tau$ (CH), 1328 $\omega + \tau$ (CH), 1309 $\omega + \tau$ (CH), 1289 $\omega + \tau$ (CH), 1266 $\omega + \tau$ (CH), 1244 $\omega + \tau$ (CH), 1220 $\omega + \tau$ (CH), 1195 $\omega + \tau$ (CH), 1172 ν(C-O), 729 $\rho(CH_2)$, 719 $\rho(CH_2)$ cm^{-1}	(Derrick et al., 1999; Maia et al., 2013)
Elemi	1687 ν(C=C), 1589 $\delta(CH_2)$, 1455 $\delta(CH_2)$, 1378 $\delta(CH_3)$, 1240 δ(COH), ν(C-O), 1129 ν(C-O), 1035 δ(COH), ν(C-O), 993, 911 cm^{-1}	(Derrick et al., 1999; Heidrich et al., 2024)
Spike oil	1735 ν(C=O), 1450 overlap of CH_2 deformation and asymmetrical CH_3 deformation, 1375, 1306, 1270, 1234, 1215, 1167, 1080, 1053, 1018, 984, 919, 841 cm^{-1}	(Agatonovic-Kustrin et al., 2020)
Calcite (RRUFF)	1414 $\nu(CO_3^-)$, 871 δ(O-C-O), 711 cm^{-1}	(Derrick et al., 1999)
Gypsum (RRUFF)	3400 $\nu_s + \nu_{as}$(O-H), 1620, 1110 $\nu_{as}(SO_4^{2-})$, 667 cm^{-1}	
Talc (RRUFF)	3673, 1000, 664 cm^{-1}	
Barite (RRUFF)	1169 $\nu_{as}(SO_4^{2-})$, 1067, 981, 631, 605 cm^{-1}	

[illegible]

δ ^{1}H/ppm (multiplicity, J /Hz)	group	functional group	Assignment (compound - binder or solvent)
6.41 (s)	HR-	aromatic protons	elemicin - elemi
5.91 (dd, 17.3, 10.8)	$RCH=CH_2$	vinyl proton	linalool - lavender oil
5.40-5.30 (m)	RCH=CHR	vinyl proton	unsaturated fatty acid - beeswax
5.21 (dd, 1.3)	HCH=CHR	vinyl proton	linalool – lavender oil
5.18 (t, 3.5)	$RCH_2CH=CR$	vinyl proton	β amyrin – copal/elemi
5.12 (t, 3.6)	RCH2CH=CR	vinyl proton	α amyrin – copal/elemi
5.06 (dd, 1.3)	HCH=CHR	vinyl proton	linalool - lavender oil
4.90 (m)	$CH_3CHO(COR)$ R	ester ω-1 alcohol	ester – beeswax
4.80 (m)	RCHO(COR) R	ester 2°alcohol	ester – beeswax
4.68 (d, 2.5)	$HCH=CCH_3R$	vinyl proton	lupeol – copal/elemi
4.56 (m)	$HCH=CCH_3R$	vinyl proton	lupeol – copal/elemi
4.05 (t, 6.7)	RCH2O(COR)	ester 1°alcohol	ester – beeswax
3.85 (s)	CH_3OR	methoxy (meta)	elemicin – elemi
3.82 (s)	CH_3OR	methoxy (para)	elemicin – elemi
3.79 (m)	CH_3CHOHR	ω-1 alcohol	alcohol – beeswax
3.64 (t, 6.7)	RCH_2OH	1° alcohol	alcohol - beeswax
3.53 (m)	RCHOHR	2° alcohol	alcohol - beeswax
3.38 (t, 2.9)		Unknown	copal
3.22 (dd, 11.4, 4.8)	RCHOHR	2° alcohol	lupeol – copal/elemi
2.34 (t, 7.5)	RCH2COOH	carboxylic acid	free fatty acids - beeswax
2.28 (t, 7.5)	RCH2COOR	Ester	ester - beeswax
2.01 (m)	RCH2CH=CHCH2R	double bond	unsaturated fatty acid - beeswax
2.00 (s)	R=CHCH2CH2R	double bond	linalool - lavender oil
1.68 (d, 1.4)	$CH_3CCH_3=R$	double bond	linalool - lavender oil
1.67 (s)	$CH_3CR=$	methyl α – vinyl	lupeol – copal/elemi
1.61 (m)	RCH2CH2COO-	ester-carboxylic acid	ester - beeswax
1.60 (d, 1.5)	$CH_3CCH_3=R$	double bond	linalool
1.57 (m)	RCH_2CH_2OH	Alcohol	alcohol - beeswax
1.52 (d, 3.8)		Unknown	copal
1.25 (m)	RCH_2R	Methylene	alkanes - beeswax
1.19 (d, 3.1)	CH_3CHOHR	methyl ω -1 alcohol	alcohol – beeswax
0.88 (t, 6.9)	RCH_3	Methyl	alkanes - beeswax

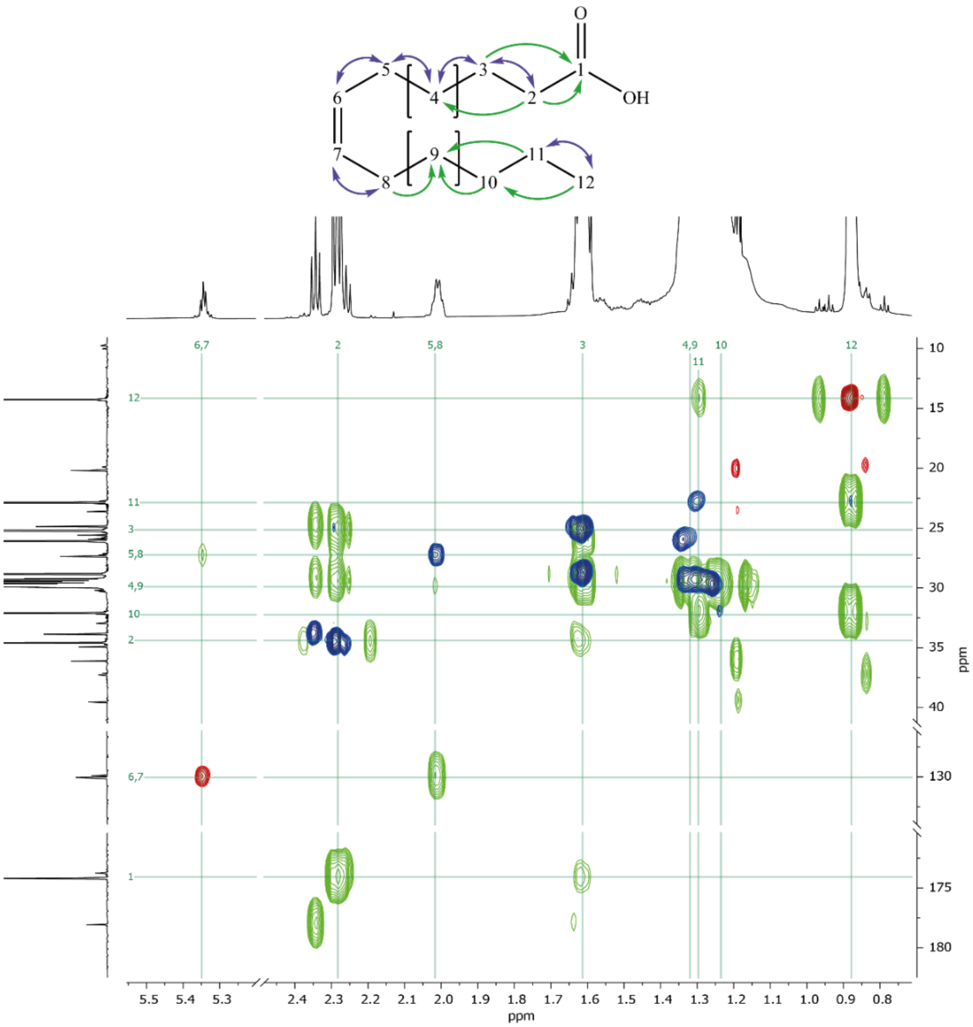

[illegible]

[illegible]

Label	δ_H /ppm	δ_C /ppm	HMBC (H→C)	COSY (H→H)
1	-	174.09	-	-
2	2.28 (t, 7.55)	34.38	C-1, 3, 4	H-3
3	1.61 (q, 6.62)	25.14	C-1, 2, 4,5	H-2, 4
4	1.32	29.91	-	H-3, 5
5	2.02	27.23	C-4, 6, 7	H-4, 6
6	5.35 (t, 4.43)	130.04	C-5, 8	H-5
7	5.35 (t, 4.43)	130.04	C-5, 8	H-8
8	2.02	27.23	C-6, 7, 9	H-7
9	1.32	29.91	-	-
10	1.24	32.23	C-9, 11	-
11	1.30	22.86	C-9, 10, 12	H-12
12	0.88 (t, 7.08)	14.15	C-10, 11	H-11

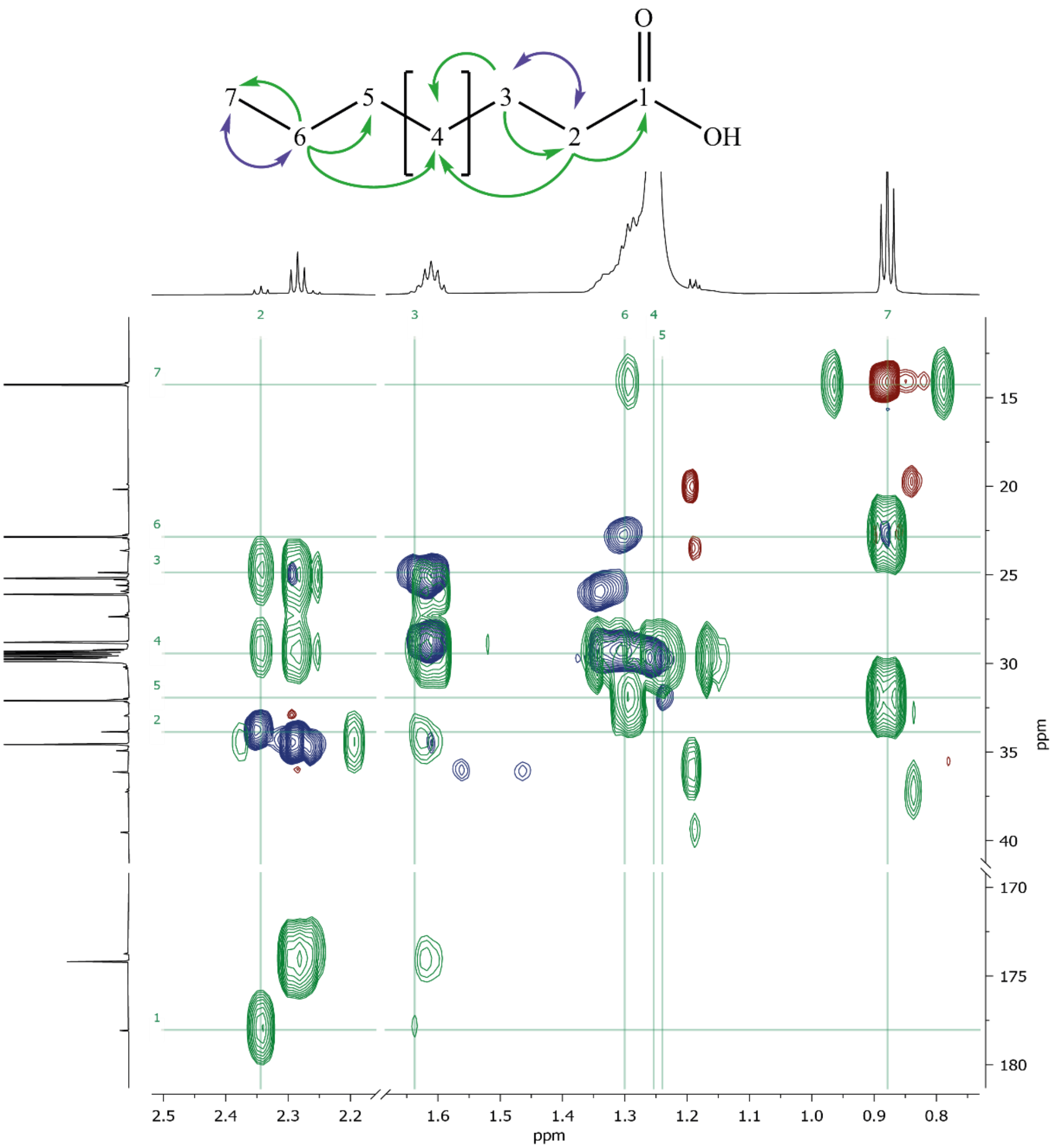

[illegible]

[illegible]

Label	δ_H /ppm	δ_C /ppm	HMBC (H→C)	COSY (H→H)
1	-	178.05	-	-
2	2.34 (t, 7.53)	33.88	C-1, 3, 4	H-3
3	1.64 (q, 7.59)	24.86	C-4, 2	H-2, 4
4	1.25	29.43	-	H-3
5	1.24	31.93	C-4	-
6	1.30	22.85	C-4, 5, 7	H-7
7	0.88 (t, 7.08)	14.27	C-5, 6	H-6

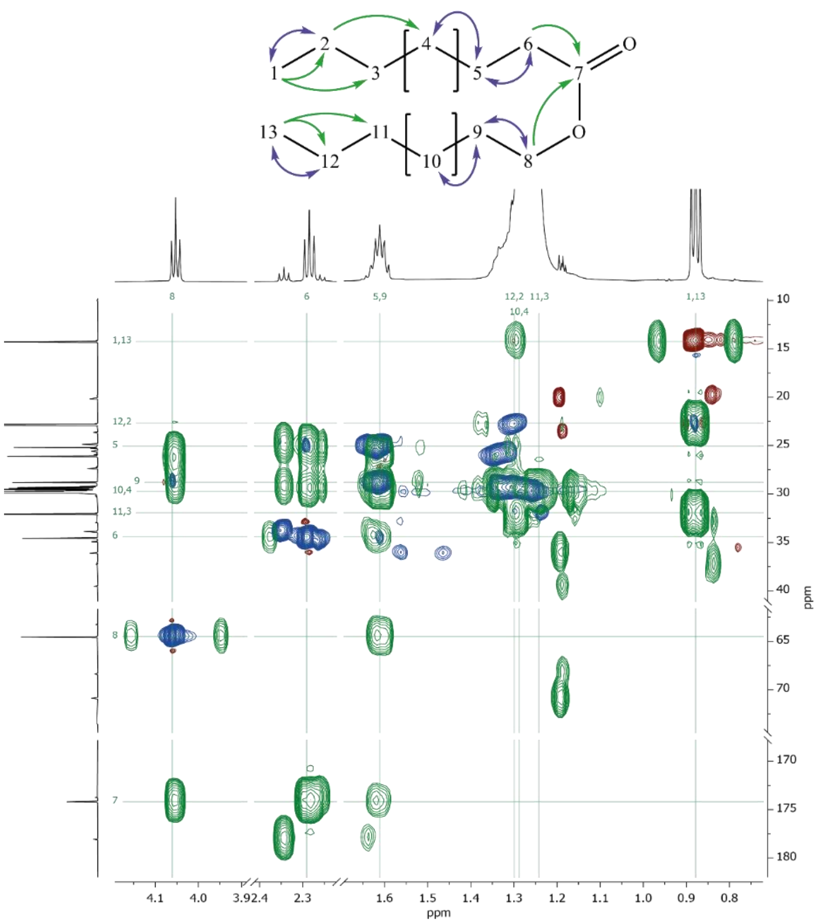

[illegible]

[illegible]

Label	δH /ppm	δC /ppm	HMBC (H→C)	COSY (H→H)
1	0.88 (t, 7.08)	14.26	C-2, 3	H-2
2	1.30	22.66	C-1, 3, 4	H-1
3	1.24	31.94	C-4	-
4	1.29	29.74	C-3, 5	H-5
5	1.61 (q, 6.62)	25.05	C-4, 7	H-4,6
6	2.28 (t, 7.55)	34.43	C-4, 5, 7	H-5
7	-	174.19	-	-
8	4.06 (t, 4.05)	64.5	C-7, 9	H-9
9	1.61 (q, 6.62)	28.8	C-8, 10	H-8, 10
10	1.29	29.74	C-9, 11	H-9
11	1.24	31.94	C-9, 10	-
12	1.30	22.66	C-10, 11, 13	H-13
13	0.88 (t, 7.08)	14.26	C-11, 12	H-12

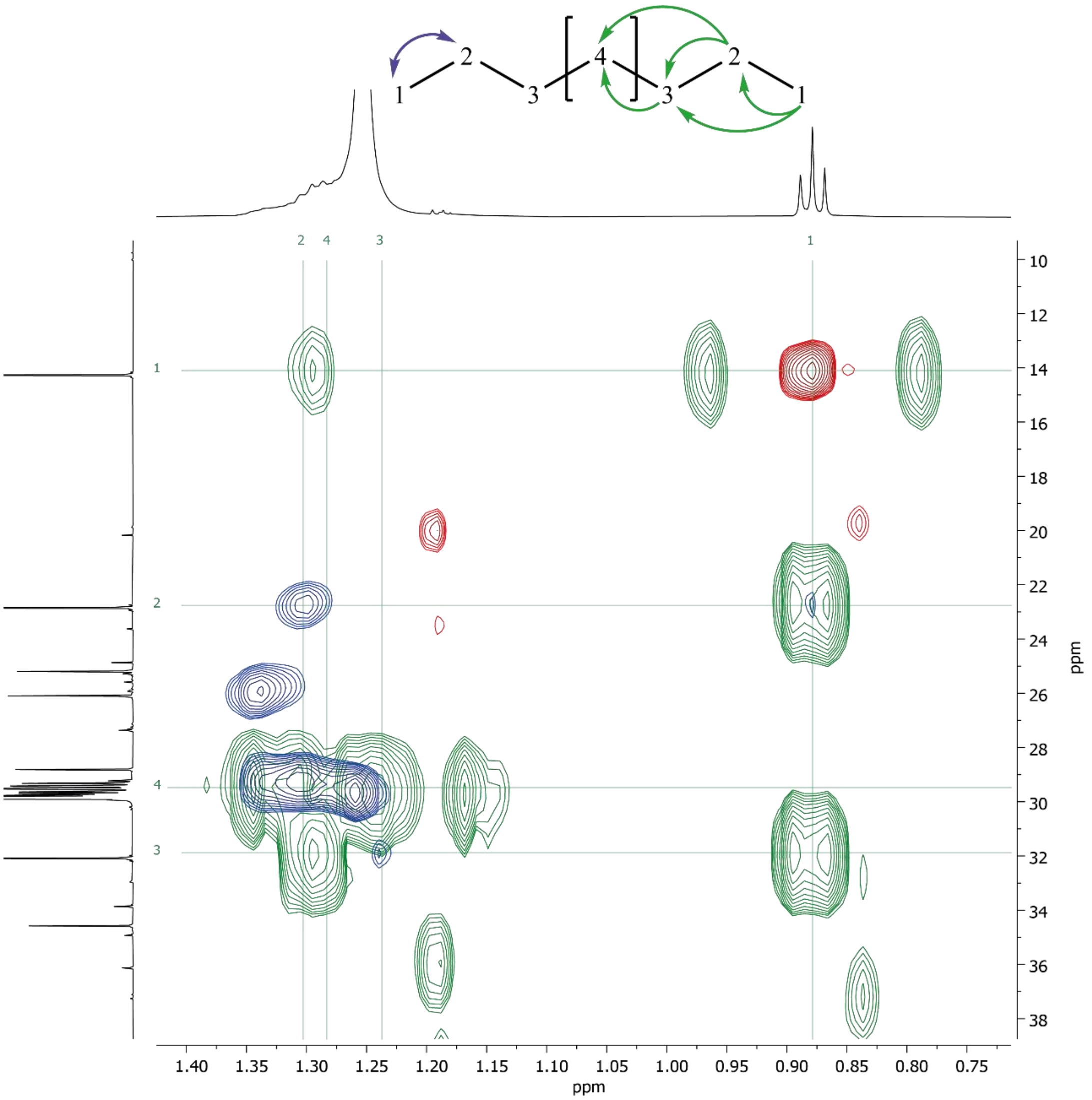

[illegible]

[illegible]

Label	δ_H /ppm	δ_C /ppm	HMBC (H→C)	COSY (H→H)
1	0.88 (t, 7.08)	14.10	C-2, 3	H-2
2	1.30	22.77	C-1, 3, 4	H-1
3	1.24	31.88	C-4	-
4	1.28	29.48	C-3	-

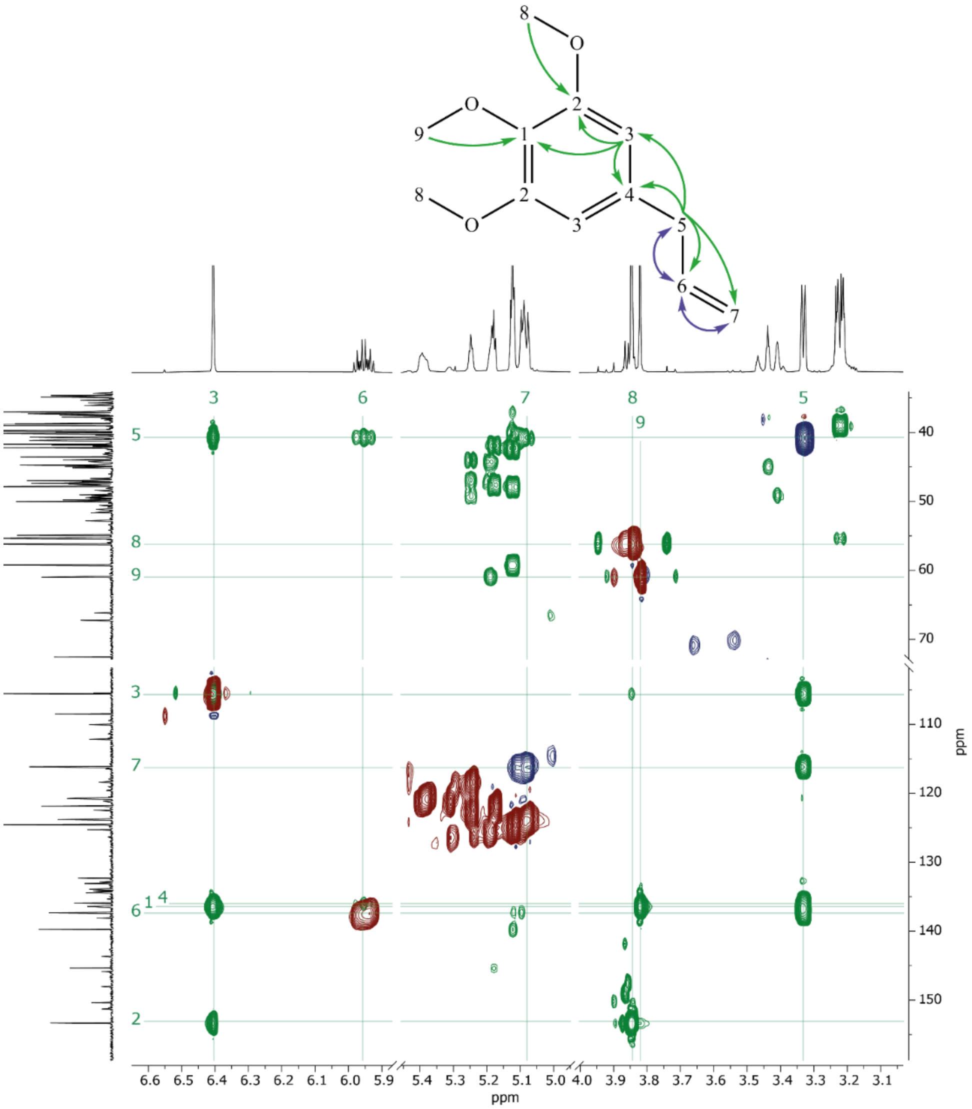

[illegible]

[illegible]

Label	δH /ppm	δc /ppm	HMBC (H→C)	COSY (H→H)
1	-	136.43	-	-
2	-	153.07	-	-
3	6.40 (s)	105.69	C-1, 2, 4, 5	-
4	-	136.1	-	-
5	3.33 (d, 6.78)	40.69	C-3, 4, 6, 7	H-6
6	5.96 (ddt 16.85, 10.03, 6.77)	137.37	C-4, 5	H-5, 7
7	5.08 (m)	116.28	C-5	H-6
8	3.84 (s)	56.22	C-2	-
9	3.82 (s)	60.99	C-1	-

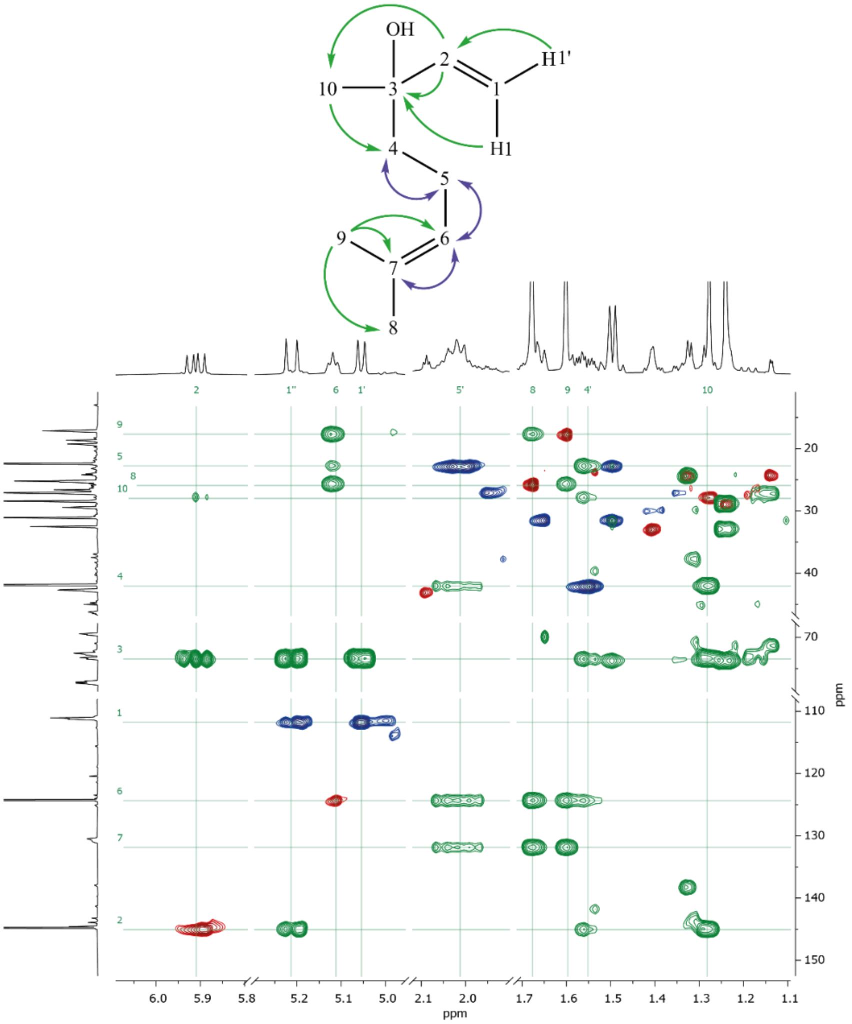

[illegible]

[illegible]

Label	δH /ppm	δc /ppm	HMBC (H→C)	COSY (H→H)
1	5.05 (d, 10.86)	111.76	C-2, 3	-
1’	5.21 (d, 17.42)	111.76	C-2, 3	-
2	5.91 (dd, 17.30, 10.82)	145.06	C-3, 10	-
3	-	73.45	-	-
4	1.55	42.05	C-2, 3, 5, 6	H-5
5	2.01 (m)	22.74	C-4, 6, 7	H-4, 5
6	5.11	124.36	C-5, 8, 9	H-5f
7	-	131.85	-	-
8	1.68	25.92	C-6, 7, 9	-
9	1.60 (s)	17.67	C-6, 7, 8	-
10	1.28 (s)	27.97	C-2, 4	-

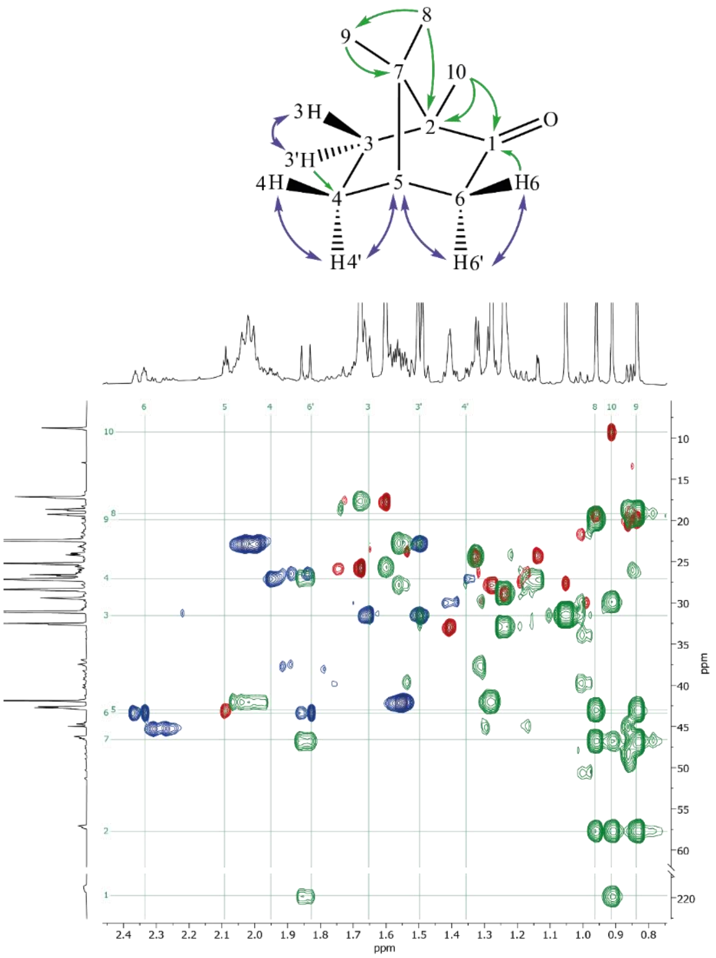

[illegible]

[illegible]

Label	δH /ppm	δC /ppm	HMBC (H→C)	COSY (H→H)
1	-	219.73	-	-
2	-	57.77	-	-
3	1.50	31.56	C-1, 4	H-3’, 4
3’	1.65	31.56	C-1, 4	H-3, 4
4	1.95	27.07	C-7	H-3, 3’, 4’, 5
4’	1.36	27.07	C-7	H-3, 3’, 4, 5
5	2.09 (t, 4.67)	43.02	C-1, 2	H-4, 4’, 5
6	2.33 (dt, 17.41, 3.94)	43.40	C-1, 7	H-5, 6’
6’	1.83	43.40	C-1, 7	H-5, 6
7	-	46.61	-	-
8	0.96 (s)	19.20	C-2, 5, 7, 9	-
9	0.84 (s)	19.82	C-2, 5, 7, 8	-
10	0.91 (s)	9.27	C-1, 2, 7	-

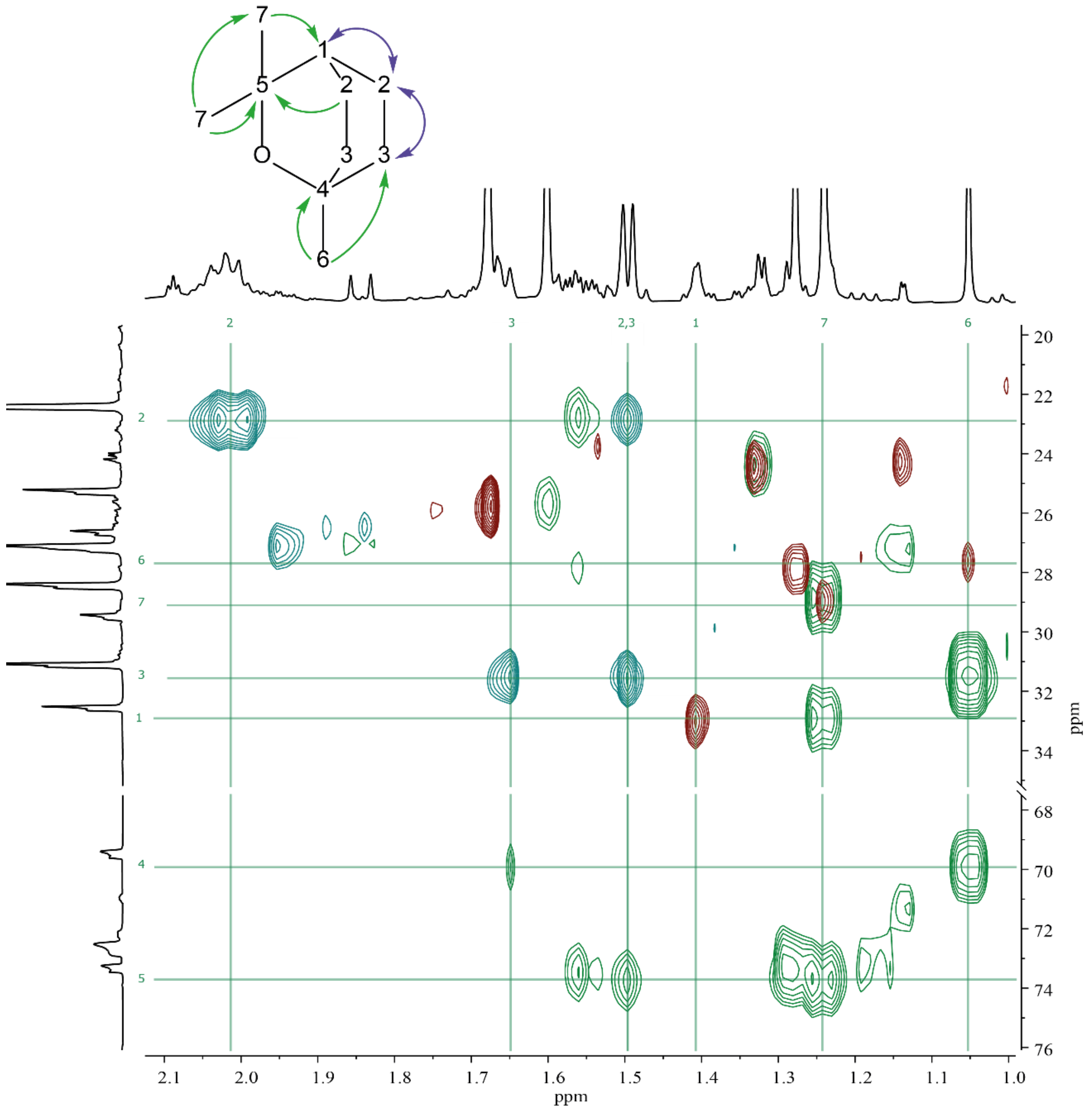

[illegible]

[illegible]

Label	δ_H /ppm	δ_C /ppm	HMBC (H→C)	COSY (H→H)
1	1.41	32.91	-	H-2
2	2.01, 1.50	22.89	C-5	H-1, 3
3	1.65, 1.50	31.56	C-4	H-2
4	-	69.93	-	-
5	-	73.71	-	-
6	1.05	27.69	C-3, 4	-
7	1.24	29.10	C-1, 5, 7	-

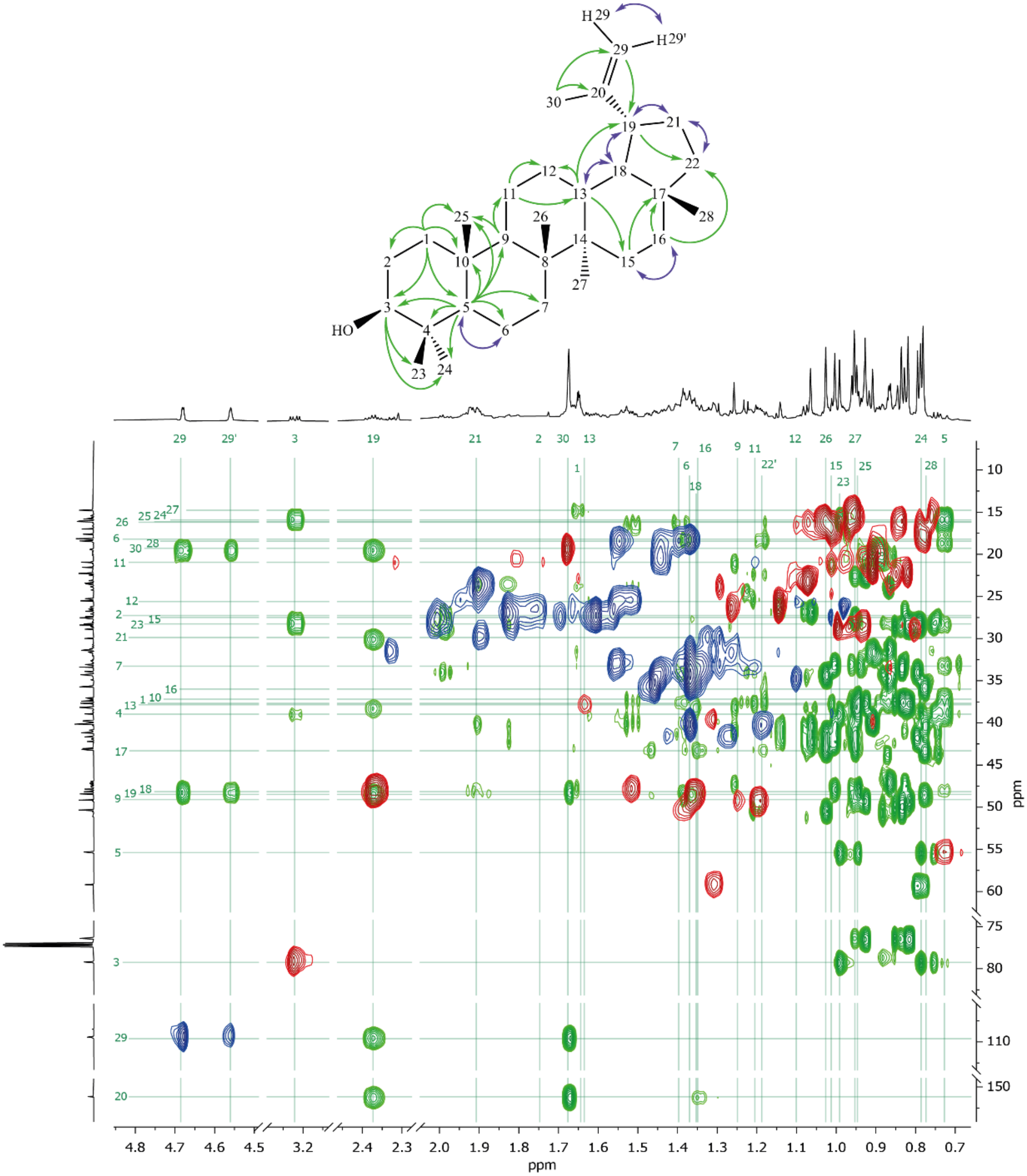
HO
ppm
ppm

[illegible]

[illegible]

Label	δ_H /ppm	δ_C /ppm	HMBC (H→C)	COSY (H→H)
1	1.67	39.82	C-2, 3, 5, 9, 10, 25	-
2	1.75	27.27	-	-
3	3.22 (dd, 11.39, 4.82)	79.26	C-23, 24, 4	-
4	-	38.97	-	-
5	0.73	55.41	C-1, 3, 4, 6, 7, 9, 10, 23, 24, 25	H-6
6	1.37	18.2	C-4, 7, 10,	H-5
7	1.4	33.27	C-5, 6, 9,	-
8	-	-	-	-
9	1.25	49.09	C-1, 10, 11, 25	-
10		37.19	-	-
11	1.21	20.94	C-9, 10, 12, 13,	-
12	1.1	25.6	-	-
13	1.64	37.84	C-12, 15, 19	H-18
14	-	-	-	
15	1.01	27.49	C-13, 17	H-16
16	1.35	35.98	C-17, 18, 22	H-15
17	-	43.32	-	
18	1.35	48.16	C-13, 17, 19, 20	H-13, 19
19	2.37 (td, 11.09, 11.05, 5.88)	48.48	C-18, 30, 21, 22, 29, 30	H-18, 21
20	-	151.16	-	-
21	1.91	29.87	C-18, 19, 20, 22	H-19,22
22	1.19	40.2	C-17, 28	H-21
23	0.99 (s)	28.26	C-3, 4, 5, 24	-
24	0.79 (s)	15.92	C-3, 4, 5, 23	-
25	0.95	16.11	C-1, 5, 9, 10,	-
26	1.03 (s)	16.19	-	-
27	0.95	14.78	C-15, 13	-
28	0.77 (s)	18.44	C16, 17, 18, 22	-
29	4.59 (dd, 2.52, 1.38)	109.62	C-19, 20, 30	H-29’
29’	4.69 (d, 2.55)	109.62	C-19, 20, 30	H-29
30	1.68	19.28	C-19, 20, 29	-

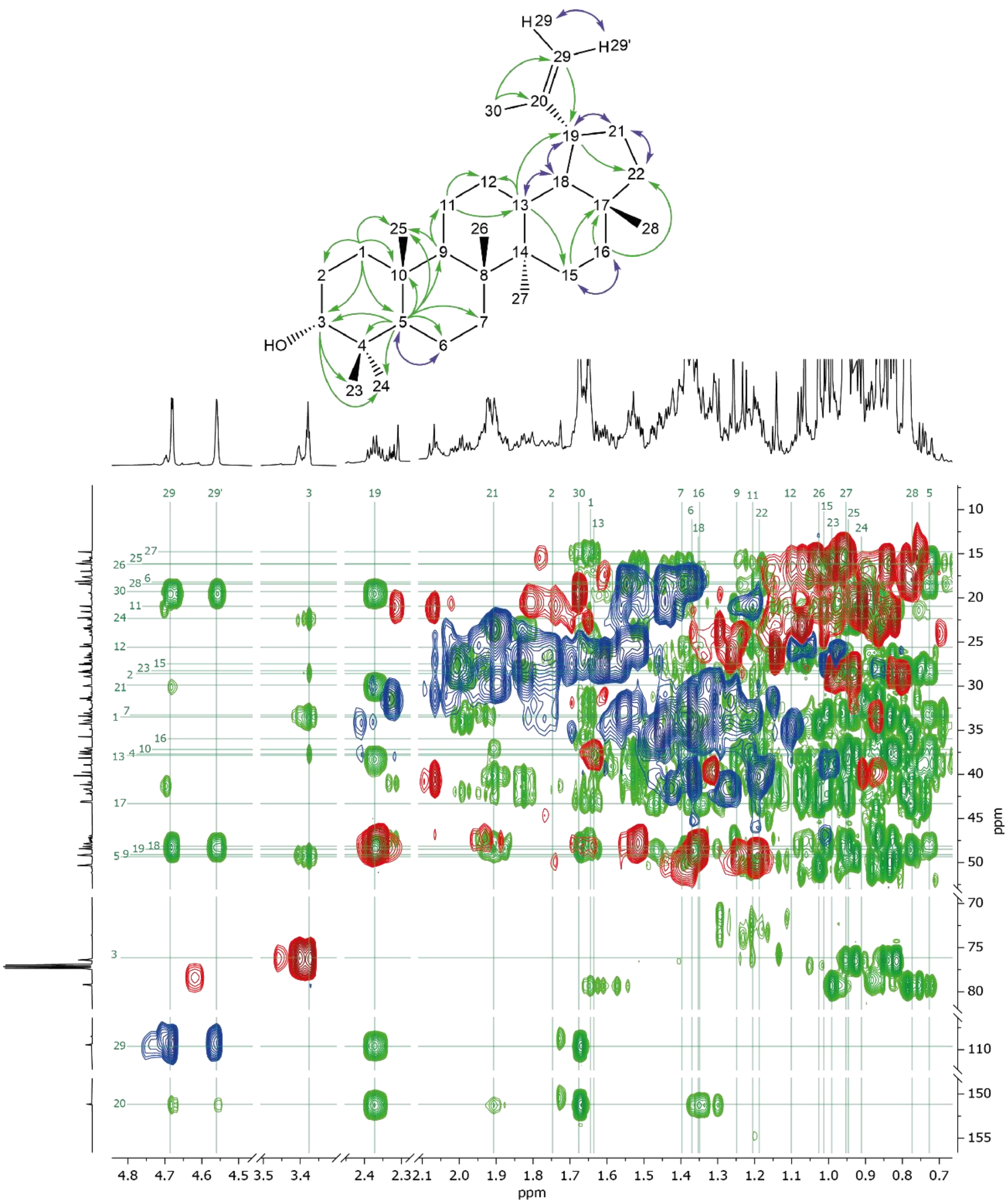

H29
H29'
HO
ppm
4.8 4.7 4.6 4.5 3.5 3.4 2.4 2.3 2.1 2.0 1.9 1.8 1.7 1.6 1.5 1.4 1.3 1.2 1.1 1.0 0.9 0.8 0.7
10 15 20 25 30 35 40 45 50 70 75 80 110 150 155

[illegible]

[illegible]

Label	δ_H /ppm	δ_C /ppm	HMBC (H→C)	COSY (H→H)
1	1.64	33.50	C-2, 3, 5, 9, 10, 25	-
2	1.75	28.56	-	-
3	3.38	76.57	C-23, 24, 4	-
4	-	37.69	-	-
5	0.73	49.33	C-1, 3, 4, 6, 7, 9, 10, 23, 24, 25	H-6
6	1.37	18.2	C-4, 7, 10,	H-5
7	1.4	33.27	C-5, 6, 9,	-
8	-	-	-	-
9	1.25	49.09	C-1, 10, 11, 25	-
10		37.19	-	-
11	1.21	20.94	C-9, 10, 12, 13,	-
12	1.1	25.6	-	-
13	1.64	37.84	C-12, 15, 19	H-18
14	-	-	-	
15	1.01	27.49	C-13, 17	H-16
16	1.35	35.98	C-17, 18, 22	H-15
17	-	43.32	-	
18	1.35	48.16	C-13, 17, 19, 20	H-13, 19
19	2.37 (td, 11.09, 11.05, 5.88)	48.48	C-18, 30, 21, 22, 29, 30	H-18, 21
20	-	151.16	-	-
21	1.91	29.87	C-18, 19, 20, 22	H-19,22
22	1.19	40.2	C-17, 28	H-21
23	0.99 (s)	28.26	C-3, 4, 5, 24	-
24	0.91	22.33	C-3, 4, 5, 23	-
25	0.95	16.11	C-1, 5, 9, 10,	-
26	1.03 (s)	16.19	-	-
27	0.95	14.78	C-15, 13	-
28	0.77 (s)	18.44	C16, 17, 18, 22	-
29	4.59 (dd, 2.52, 1.38)	109.62	C-19, 20, 30	H-29’
29’	4.69 (d, 2.55)	109.62	C-19, 20, 30	H-29
30	1.68	19.28	C-19, 20, 29	-

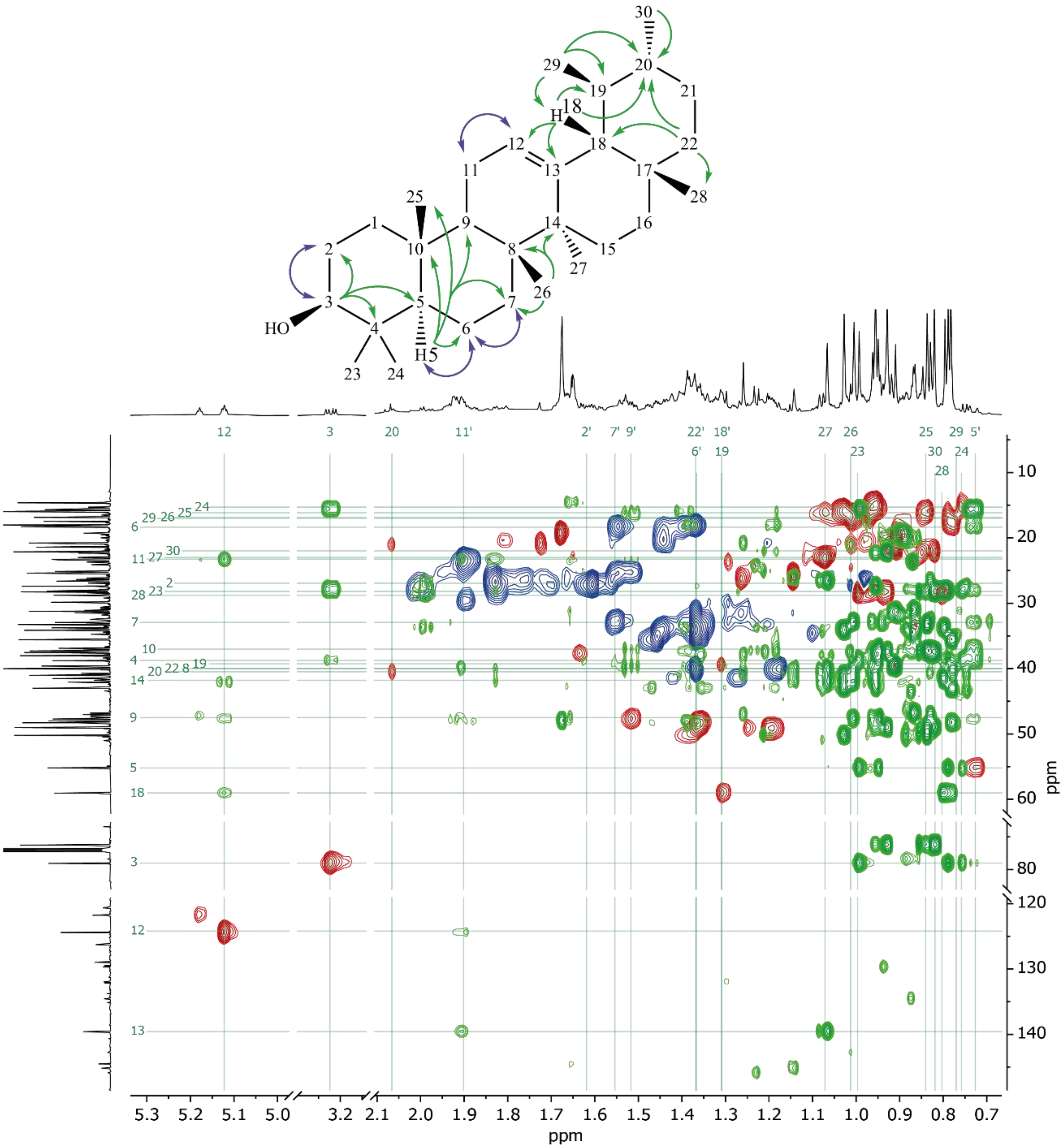
HO
ppm

[illegible]

[illegible]

Label	δ_H /ppm	δ_C /ppm	HMBC (H→C)	COSY (H→H)
1	-	-	-	-
2	1.62	26.95	C-3	H-3
3	3.22	79.02	C-2, 4, 5	H-2
4	-	38.76	-	-
5	0.73	55.19	C-3, 4, 6, 7, 9, 10, 25	H-6
6	1.37	18.36	C-4, 7, 8, 10	H-5, 7
7	1.55	32.94	C-5, 6, 14, 26	H-6
8	-	39.97	-	-
9	1.52	47.51	C-7, 8, 10, 11, 14, 25	-
10	-	37.02	-	-
11	1.9	23.25	C-8, 9, 10, 12, 13	H-12
12	5.12	124.2	C-9, 11, 13, 14	H-11
13	-	139.59	-	-
14	-	41.8	-	-
15	-	-	-	-
16	-	-	-	-
17	-	-	-	-
18	1.31	59.01	C-12, 13, 14, 19, 20	H-19
19	1.31	39.27	C-13, 18, 20, 30	H-18
20	2.07	40.49	C-22	-
21	-	-	-	-
22	1.37	40.01	C-20	-
23	1.00	28.18	C-3, 4, 5, 24	-
24	0.76	15.27	C-3, 4, 5, 23	-
25	0.84	16.15	C-5, 9, 10	-
26	1.01	16.83	C-7, 8, 14	-
27	1.07	22.99	C-8, 14	-
28	0.80	28.77	C-22	-
29	0.77	17.01	C-18, 19, 20	-
30	0.82	21.98	C-19, 20	-

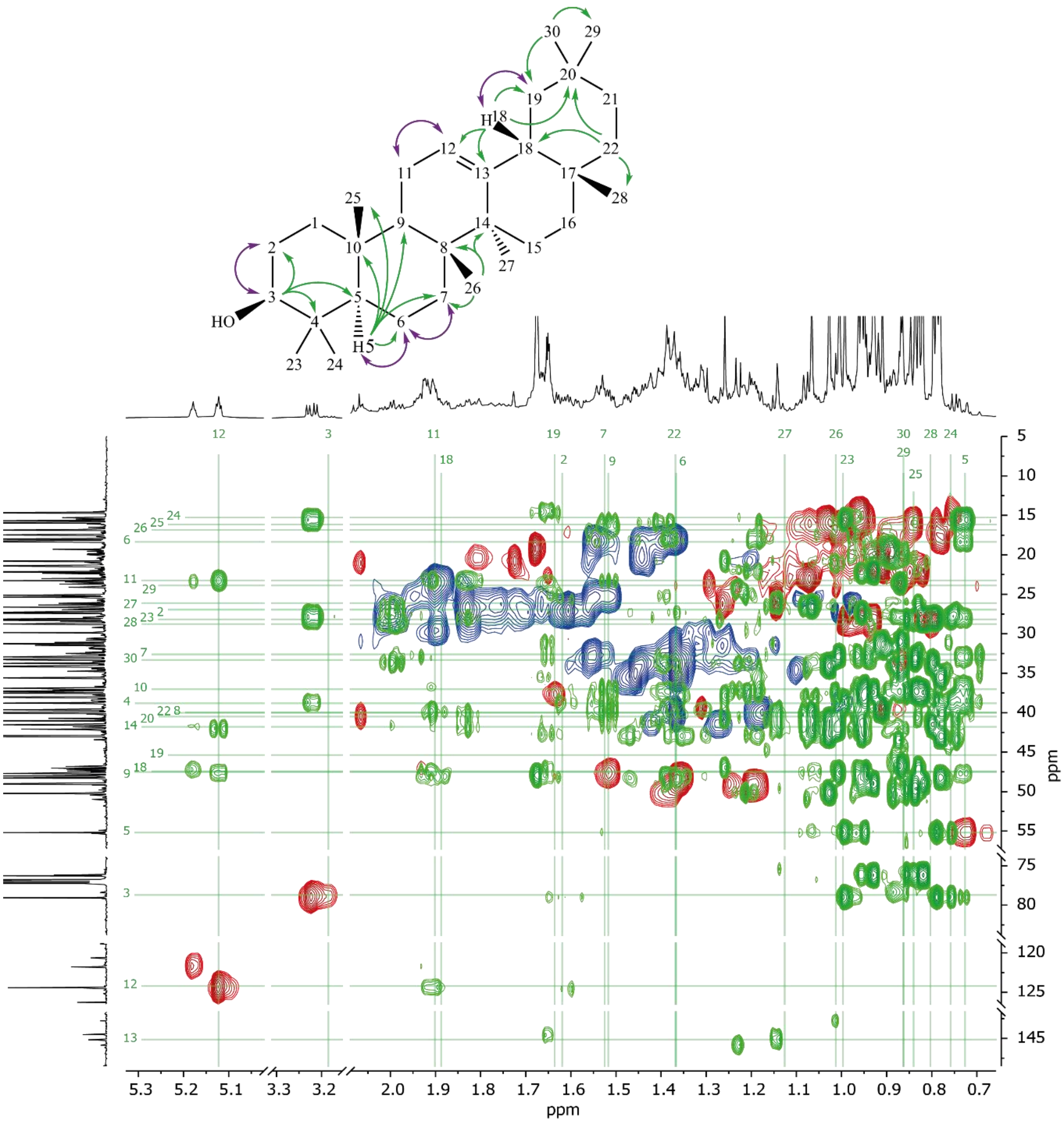
HO
ppm
ppm

[illegible]

[illegible]

Label	δ_H /ppm	δ_C /ppm	HMBC (H→C)	COSY (H→H)
1	-	-	-	-
2	1.62	26.95	C-3	H-3
3	3.18	78.71	C-2, 4, 5	H-2
4	-	38.81	-	-
5	0.73	55.19	C-3, 4, 6, 7, 9, 10, 25	H-6
6	1.37	18.36	C-4, 7, 8, 10	H-5, 7
7	1.53	32.6	C-5, 6, 14, 26	H-6
8	-	39.97	-	-
9	1.52	47.51	C-7, 8, 10, 11, 14, 25	-
10	-	37.02	-	-
11	1.9	23.25	C-8, 9, 10, 12, 13	H-12
12	5.12	124.2	C-9, 11, 13, 14	H-11
13	-	145.15	-	-
14	-	41.8	-	-
15	-	-	-	-
16	-	-	-	-
17	-	-	-	-
18	1.89	47.43	C-12, 13, 14, 19, 20	H-19
19	1.64	45.37	C-13, 18, 20, 29, 30	H-18
20	-	40.49	-	-
21	-	-	-	-
22	1.37	40.01	C-18, 20, 28	-
23	1	28.18	C-3, 4, 5, 24	-
24	0.76	15.27	C-3, 4, 5, 23	-
25	0.84	16.15	C-5, 9, 10	-
26	1.01	16.83	C-7, 8, 14	-
27	1.13	26.12	C-8, 14	-
28	0.8	28.77	C-22	-
29	0.86	23.88	C-19, 30	-
30	0.86	33.32	C-19, 29	-

[illegible]

rt	Compound	CAS	Rivera's Mock-up	Cauterized mock-up	M28	M29N	M29V
5.30	cis-Linaloloxide	1000121-97-4	-	-	-	+	+
5.42	Linalool	78-70-6	-	-	-	+	-
6.18	(+)-2-Bornanone	464-49-3	-	-	+	+	+
6.48	endo-Borneol	507-70-0	-	-	-	+	+
11.63	Elemicin	639-99-6		-	-	-	-
12.74	Isoelemicin	487-12-7	+	-	-	-	-
14.44	α-Phellandrene, dimer	7350-11-0	+	-	+	+	+
14.68	Cryptomeridiol	1000411-50-2	+	+	-	-	-
21.50	Tetracosane	646-31-1	+	+	-	-	-
22.72	Pentacosane	629-99-2	+	+	-	-	-
23.92	Hexacosane	630-01-3	+	+	-	-	-
25.10	Heptacosane	593-49-7	+	+	-	-	-
26.25	Octacosane	630-02-4	+	+	-	-	-
27.37	Nonacosane	630-03-5	+	+	-	-	-
28.58	Triacontane	638-68-6	+	+	-	-	-
29.99	Hentriacontane	630-04-6	+	+	-	-	-
34.41	β-Amyrin acetate	1616-93-9	+	+	+	+	+
34.91	β-Amyrin	559-70-6	+	+	-	-	-
35.49	Lupeol	545-47-1	+	+	+	+	+
36.01	α-Amyrin	638-95-9	+	+	+	+	+

Notation: + indicates that the compound was identified in the sample; - indicates that the compound was not identified

[illegible]

rt	Compound	CAS	Rivera's mock-up	Cauterized mock-up	M28	M29N	M29V
13.31	Phthalic acid, 2TMS derivative	2078-22-0	-	-	+	+	+
13.84	Tridecanoic acid, TMS derivative	169597-14-2	+	+	+	+	+
17.19	Palmitic Acid, TMS derivative	55520-89-3	+	+	+	+	+
19.30	Oleic Acid, (Z)-, TMS derivative	21556-26-3	+	-	-	-	-
19.61	Stearic acid, TMS derivative	18748-91-9	+	+	-	-	-

Notation: + indicates that the compound was identified in the sample; - indicates that the compound was not identified

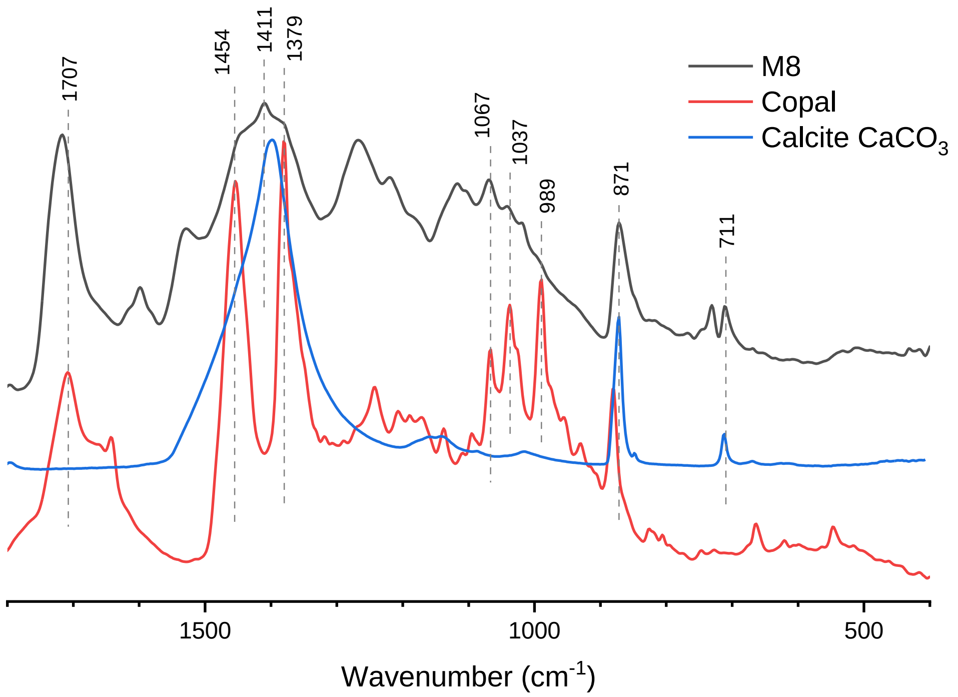
1707
1454
1411
1379
1067
1037
989
871
711
M8
Copal
Calcite CaCO$_3$
1500
1000
500
Wavenumber (cm^{-1})

[illegible]

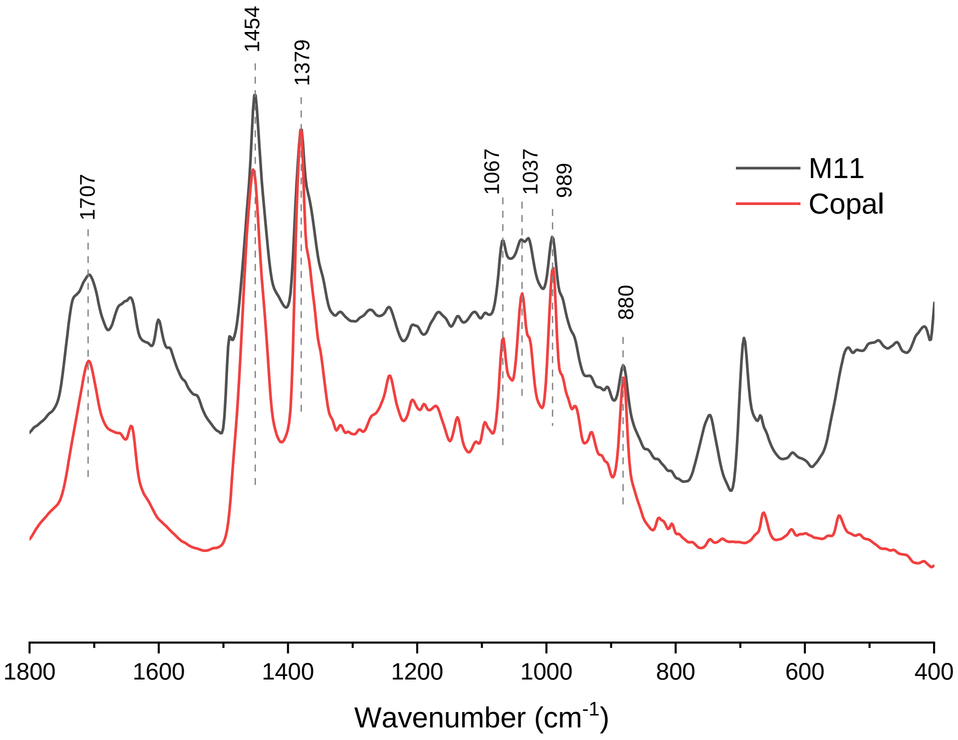
1454
1379
1707
1067
1037
989
880
M11
Copal
1800
1600
1400
1200
1000
800
600
400
Wavenumber (cm^{-1})

[illegible]

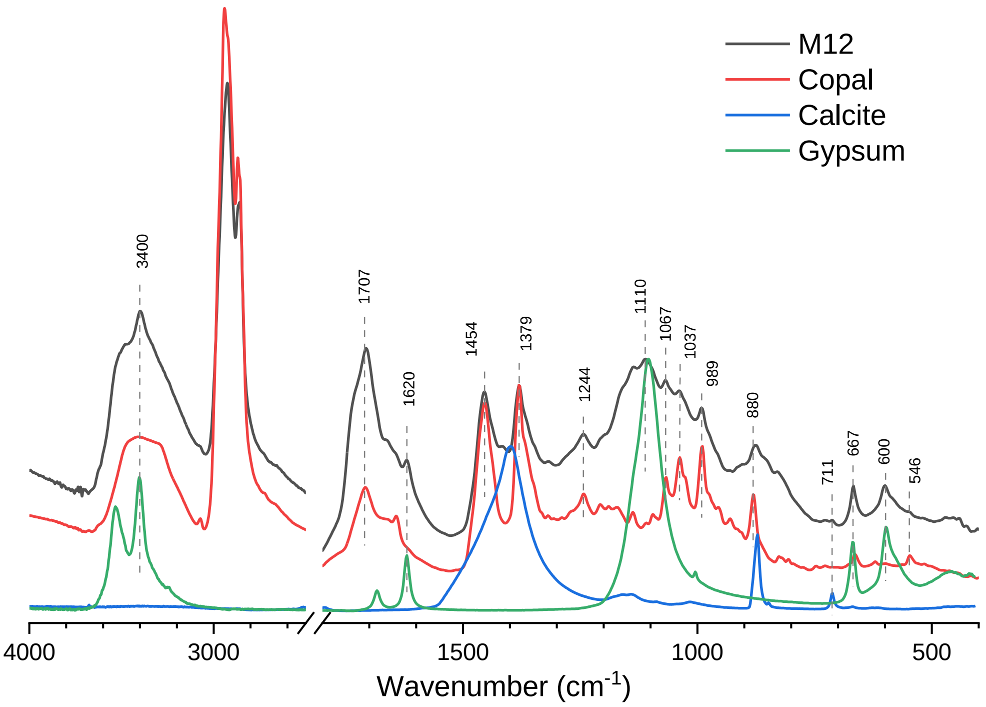
M12
Copal
Calcite
Gypsum
3400
1707
1620
1454
1379
1244
1110
1067
1037
989
880
711
667
600
546
4000
3000
1500
1000
500
Wavenumber (cm-1)

[illegible]

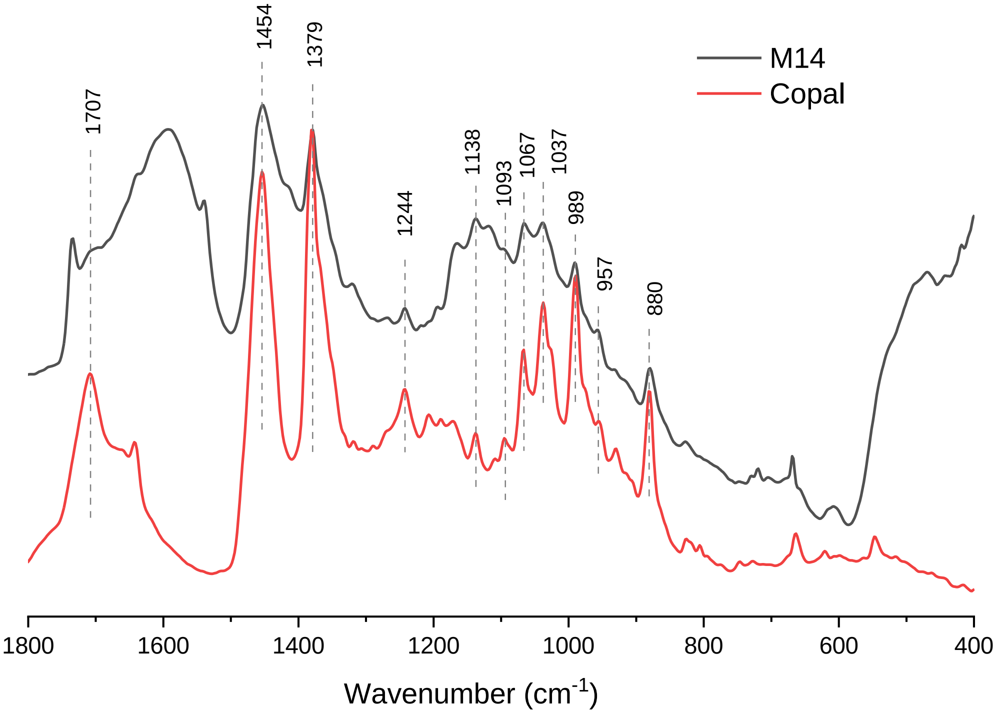
M14
Copal
1707
1454
1379
1244
1138
1093
1067
1037
989
957
880
1800
1600
1400
1200
1000
800
600
400
Wavenumber (cm-1)

[illegible]

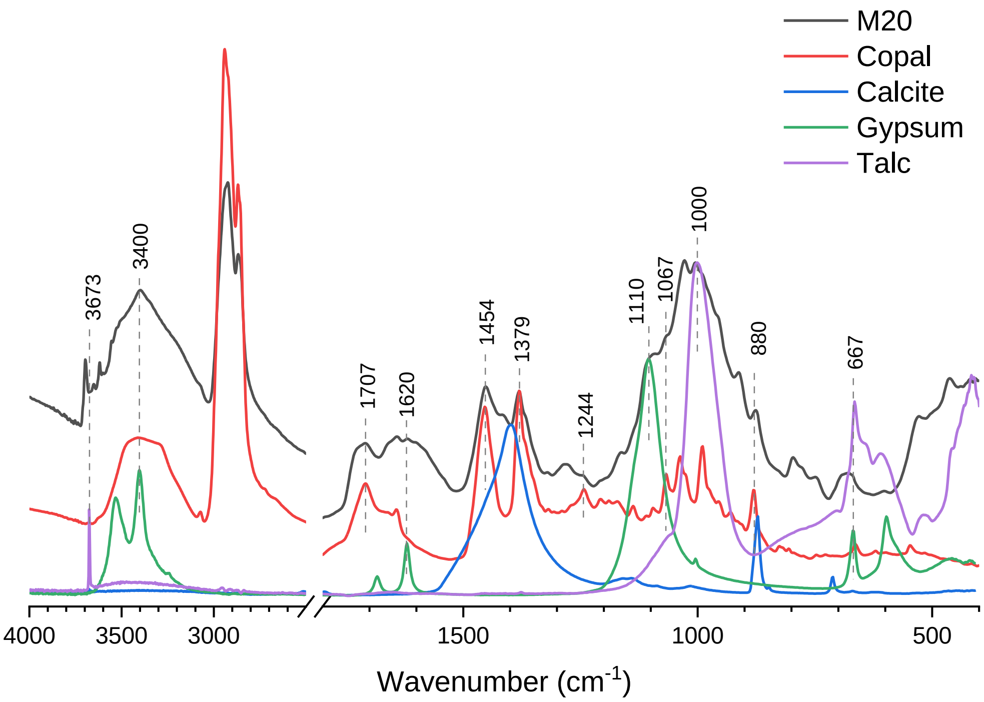
M20
Copal
Calcite
Gypsum
Talc
3673
3400
1707
1620
1454
1379
1244
1110
1067
1000
880
667
4000
3500
3000
1500
1000
500
Wavenumber (cm-1)

[illegible]

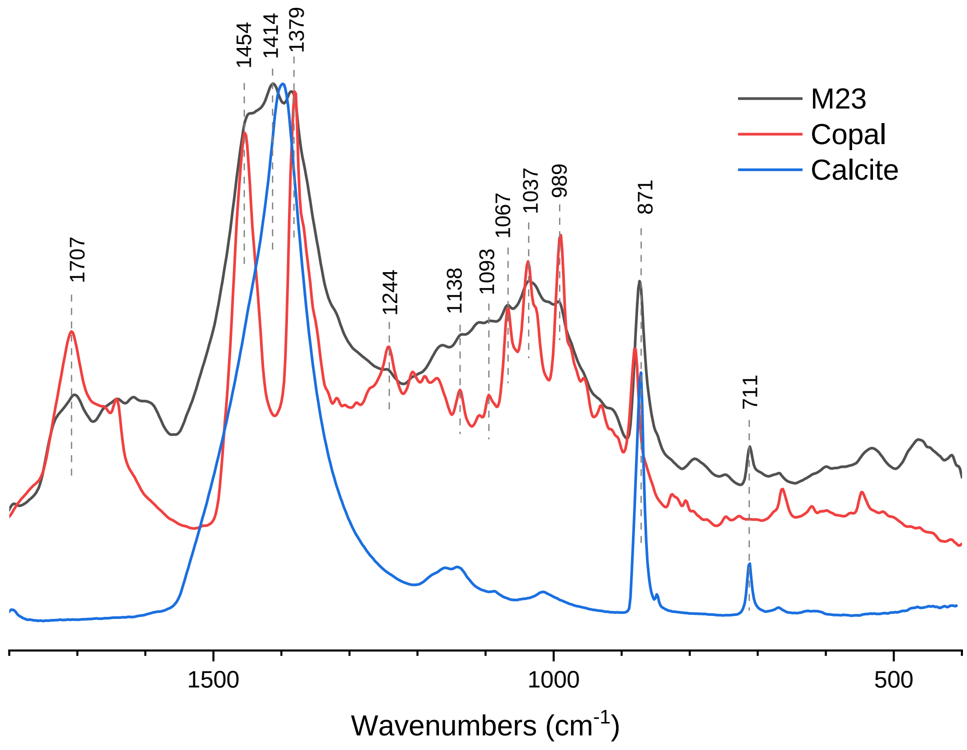
M23
Copal
Calcite
1454
1414
1379
1707
1244
1138
1093
1067
1037
989
871
711
1500
1000
500
Wavenumbers (cm-1)

[illegible]

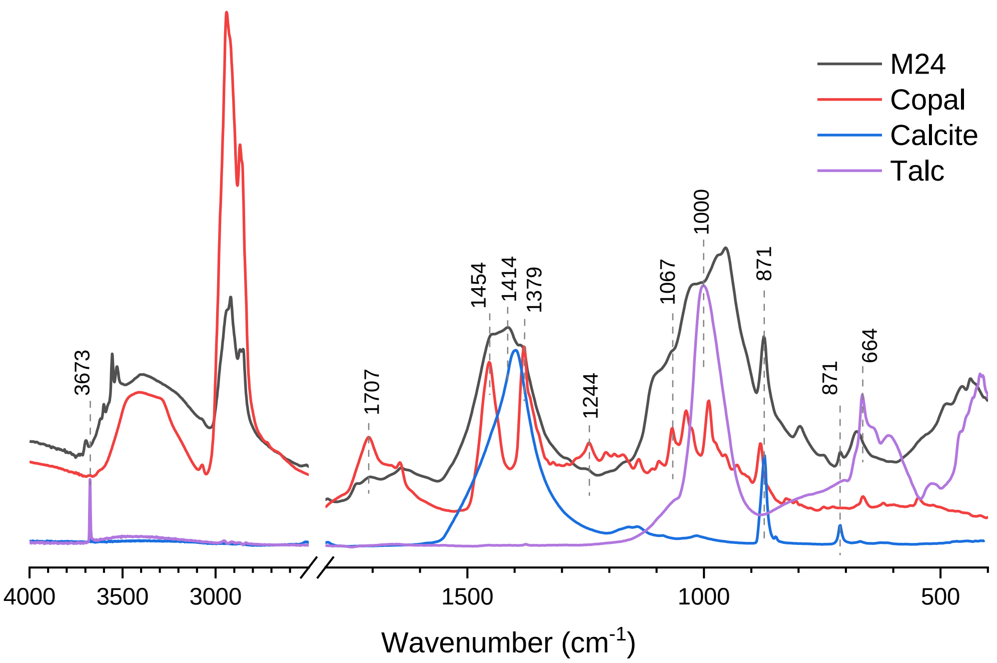
M24
Copal
Calcite
Talc
3673
1707
1454
1414
1379
1244
1067
1000
871
871
664
4000
3500
3000
1500
1000
500
Wavenumber (cm-1)

[illegible]

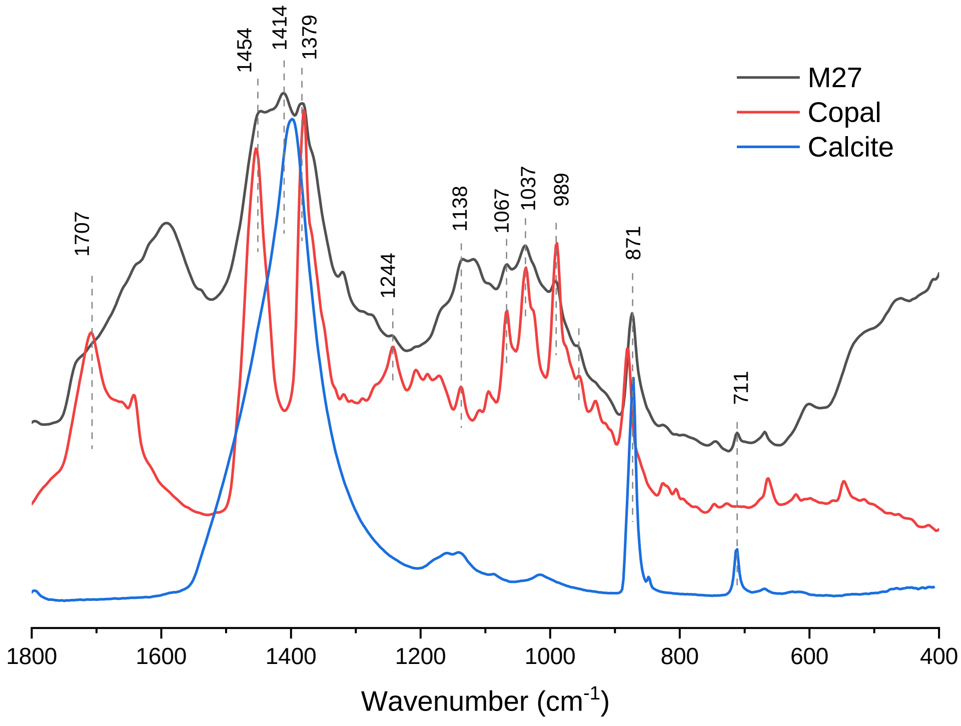
M27
Copal
Calcite
1707
1454
1414
1379
1244
1138
1067
1037
989
871
711
1800
1600
1400
1200
1000
800
600
400
Wavenumber (cm-1)

[illegible]

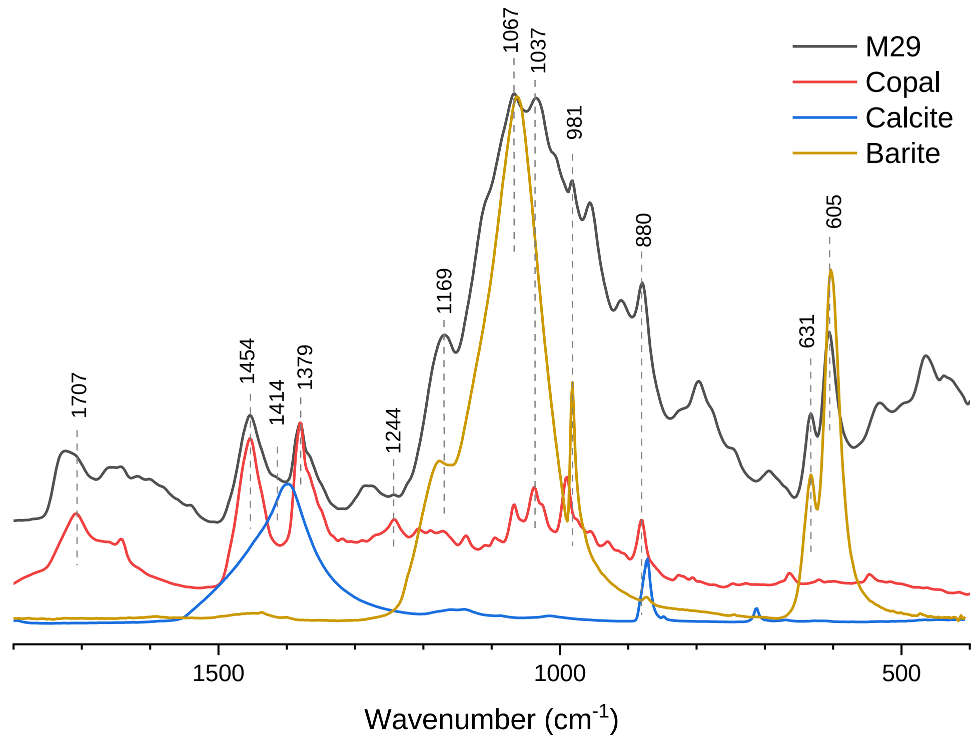
M29
Copal
Calcite
Barite
1707
1454
1414
1379
1244
1169
1067
1037
981
880
631
605
1500
1000
500
Wavenumber (cm-1)

[illegible]

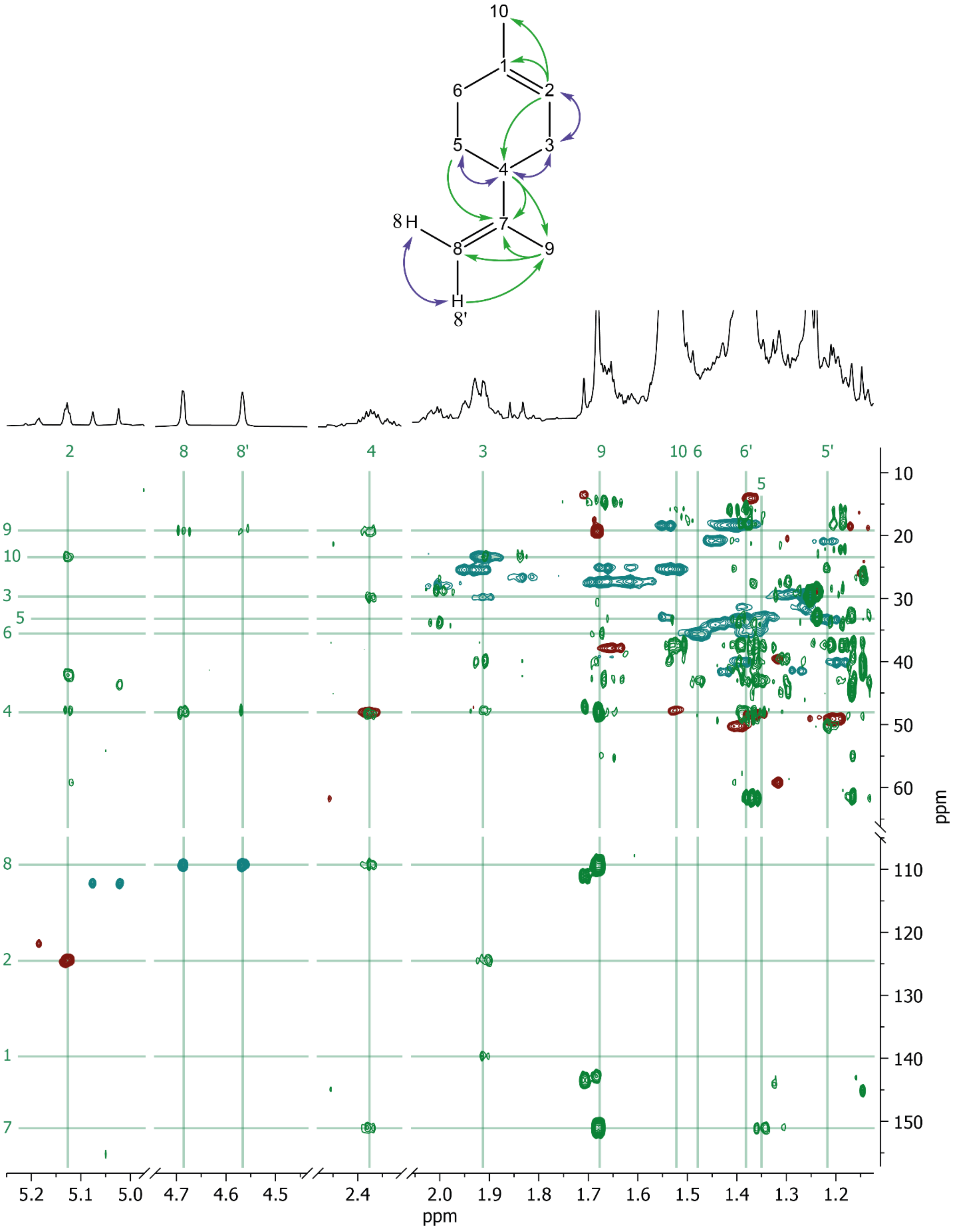
10
1
2
3
4
5
6
7
8
9
8 H
H
8'
2
8
8'
4
3
9
10
6
6'
5
5'
5.2
5.1
5.0
4.7
4.6
4.5
2.4
2.0
1.9
1.8
1.7
1.6
1.5
1.4
1.3
1.2
ppm
10
20
30
40
50
60
110
120
130
140
150
ppm

[illegible]

[illegible]

Label	δH /ppm	δC /ppm	HMBC (H→C)	COSY (H→H)
1	-	139.69	-	-
2	5.13	124.51	C-1, 10	H-3
3	1.91	29.68	C-1, 2, 4	H-2
4	2.38	48.04	C-3,7, 8, 9	H-5
5	1.35, 1.22	33.2	C-7	H-4
6	1.38, 1.48	35.57	-	-
7	-	151.11	-	-
8'	4.69	109.27	C-4,9	H-8
8	4.57	109.27	C-4,9	H8'
9	1.68	19.2	C-4,7,8	-
10	1.52	23.42	-	-

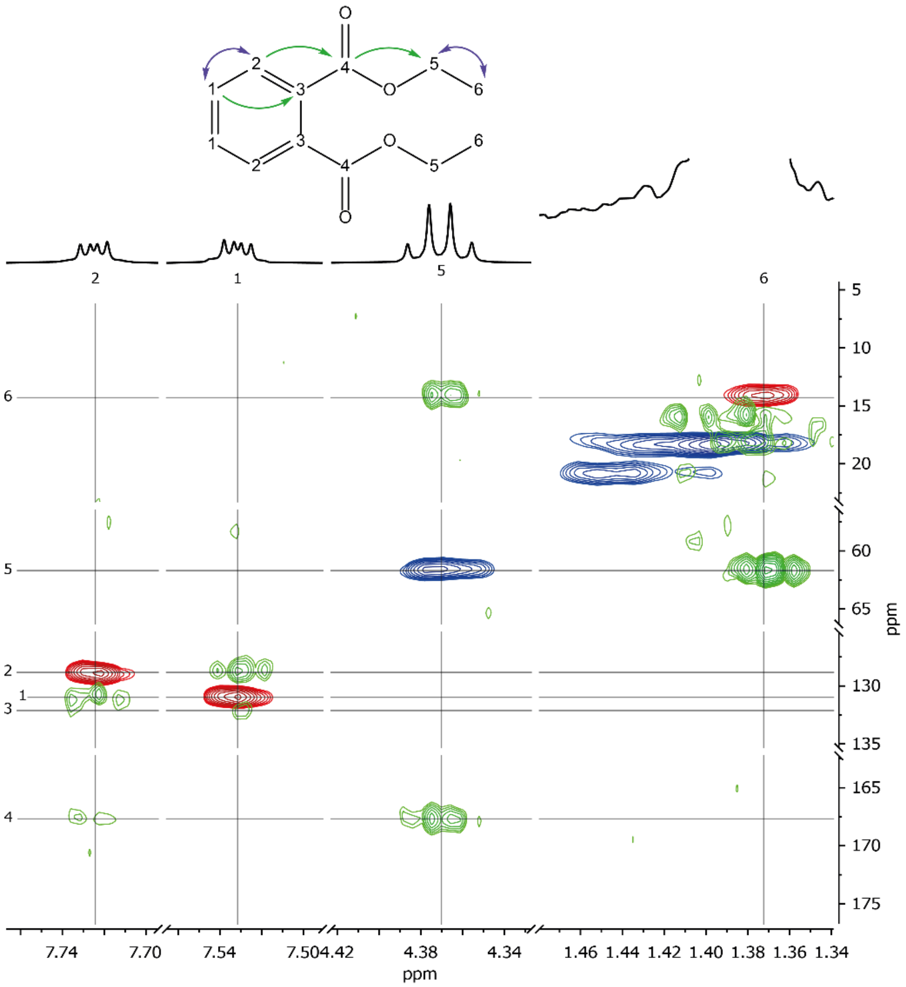

[illegible]

[illegible]

Label	δH /ppm	δC /ppm	HMBC (H→C)	COSY (H→H)

1	7.51 (dd, 5.71, 3.30)	130.97	C-2, 3	H-2
2	7.71 (dd, 5.68, 3.28)	128.83	C-1, 4	H-1
3	-	132.12	-	-
4	-	167.63	-	-
5	4.37	60.97	C-4, 6	H-6
6	1.37	14.09	C-5	H-5

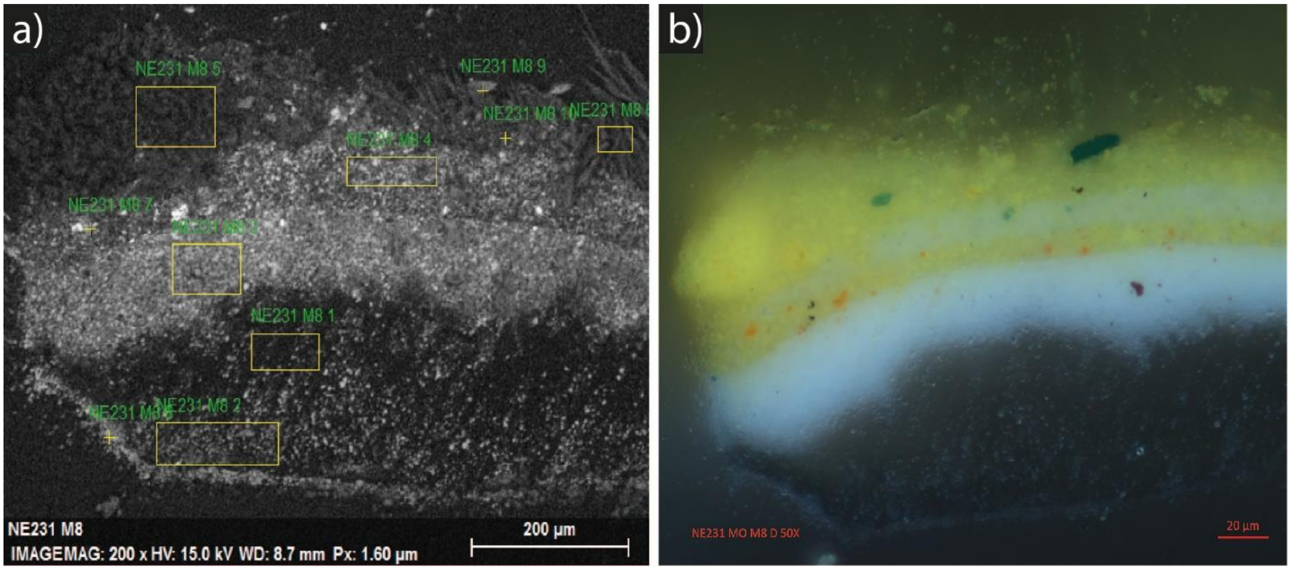

[illegible]

[illegible]

Spectrum	Mass percent (%)								
	C	O	Na	Mg	Si	S	Cr	Zn	Cd
NE231M8 1	73.00	21.04	0.18		0.06			5.71	
NE231 M8 2	68.21	23.39			0.07			8.17	
NE231 M8 3	104.78	45.26			0.06	0.14		21.46	0.33
NE231 M8 4	103.47	44.46		0.81	0.11	0.36		16.75	1.07
NE231 M8 5	61.86	30.53		2.11	0.08	0.38		3.33	1.57
NE231 M8 6	59.26	31.52	0.50	2.50	0.05	0.29		4.71	0.83
NE231 M8 7	80.80	36.64		1.96	0.20	0.66		8.91	1.52
NE231 M8 8	131.78	48.93		0.32				23.99	
NE231 M8 9	72.37	37.46	0.35	2.02	0.11	0.32	6.67	5.27	1.02

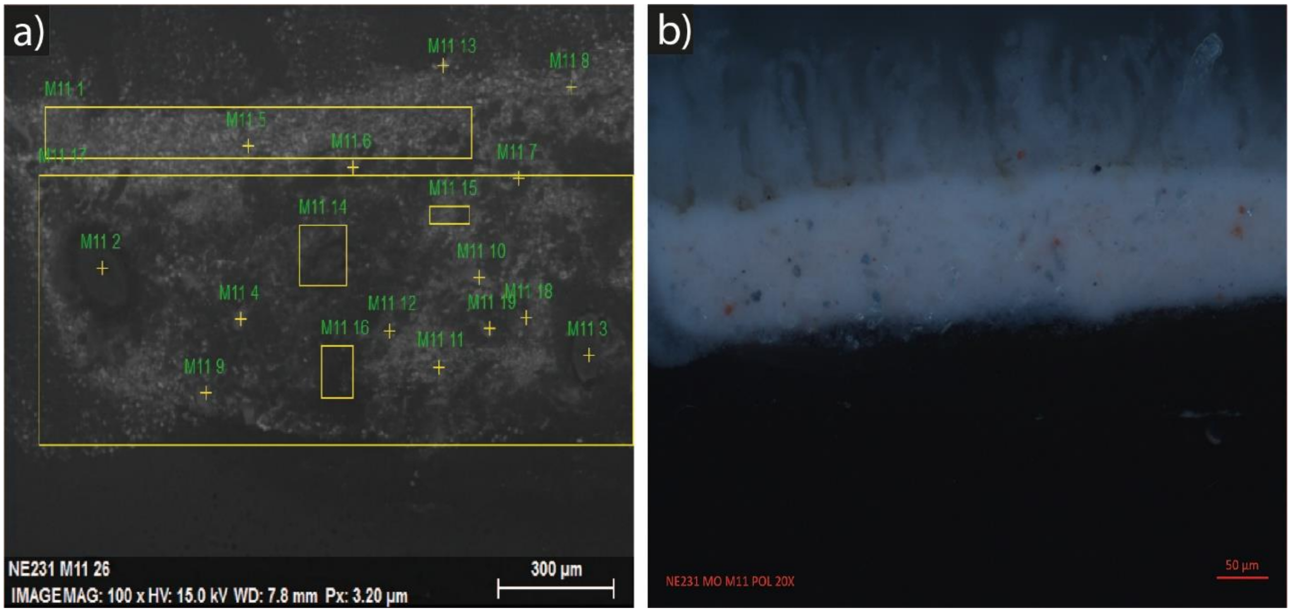

[illegible]

[illegible]

Spectrum	Mass percent (%)							
	C	O	Na	Si	S	Cl	Ti	Zn
M11 1	64.97	21.71		0.64				11.64
M11 2	70.97	24.91	0.37	0.50				3.18
M11 3	70.21	23.40	0.71	0.63	0.12	0.33	0.76	3.85
M11 4	81.13	17.56		15.44				3.55
M11 5	77.56	25.77		0.49				18.54
M11 6	81.60	18.12		16.89				4.63
M11 7	70.59	22.41		0.74				6.27
M11 8	81.88	25.21		0.41				18.02
M11 9	88.53	20.04		15.50				4.45
M11 10	85.99	17.55		18.21				5.32
M11 11	86.76	23.52	1.73	4.55				10.58
M11 12	88.91	25.57	1.85	1.35				11.38
M11 13	75.96	25.17		0.63				23.41
M11 14	69.58	22.34	0.90	1.27	0.06			5.85
M11 15	85.51	24.88		0.87				14.66
M11 16	72.97	21.82		0.69		0.24		4.28
M11 17	70.01	22.07		1.33				6.59
M11 18	78.71	21.81		0.80				12.03
M11 19	91.37	21.70	1.05	11.50				4.62

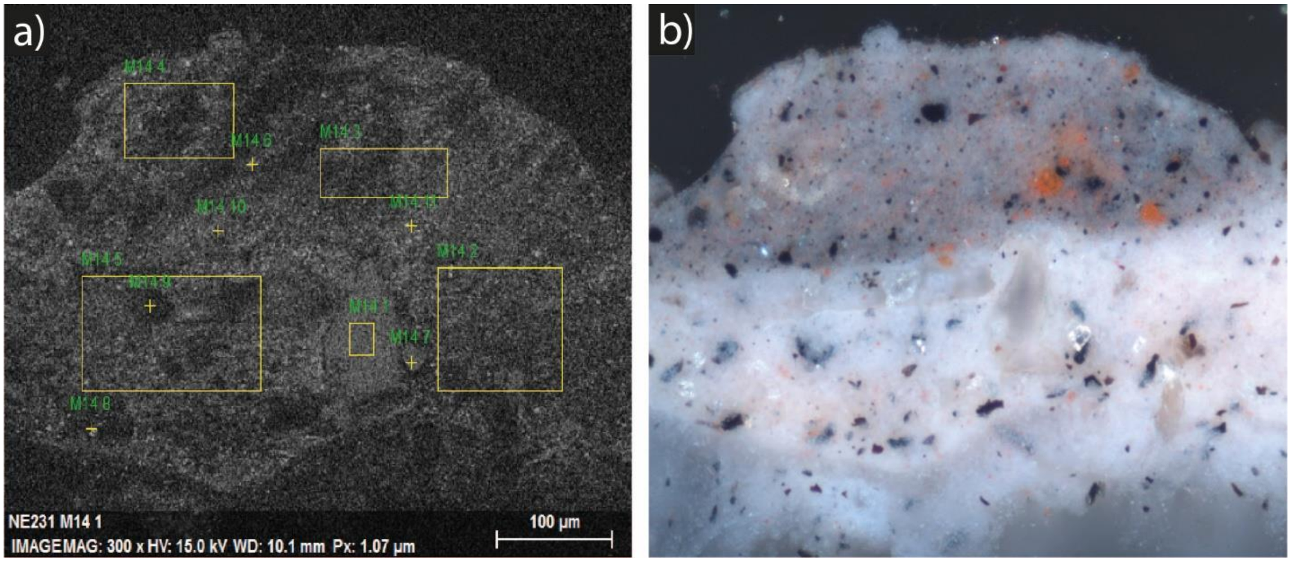

[illegible]

[illegible]

Spectrum	Mass percent (%)									
	C	O	Na	Mg	Al	Si	S	Ca	Fe	Zn
M14 1	70.88	47.00	3.53		6.10	11.47		2.41		5.52
M14 2	88.50	28.75	2.27	0.07	0.31	1.40		0.43		14.34
M14 3	67.99	23.57			0.29	1.04	0.29	0.59	0.60	11.62
M14 4	70.93	26.60			0.17	0.60		0.16		12.07
M14 5	73.21	25.09				1.19		0.27		14.18
M14 6	79.50	24.59	2.01		0.15	0.46				11.76
M14 7	95.54	22.70	1.02		0.74	28.66		0.43		4.87
M14 8	75.38	28.31			0.10	0.79		0.29	0.87	10.78
M14 9	68.38	22.02	1.29		0.11	0.54		0.20		7.45
M14 10	75.22	25.25			0.27	0.65		0.18		18.75
M14 11	71.13	25.49			0.31	0.82		0.33	3.55	12.35

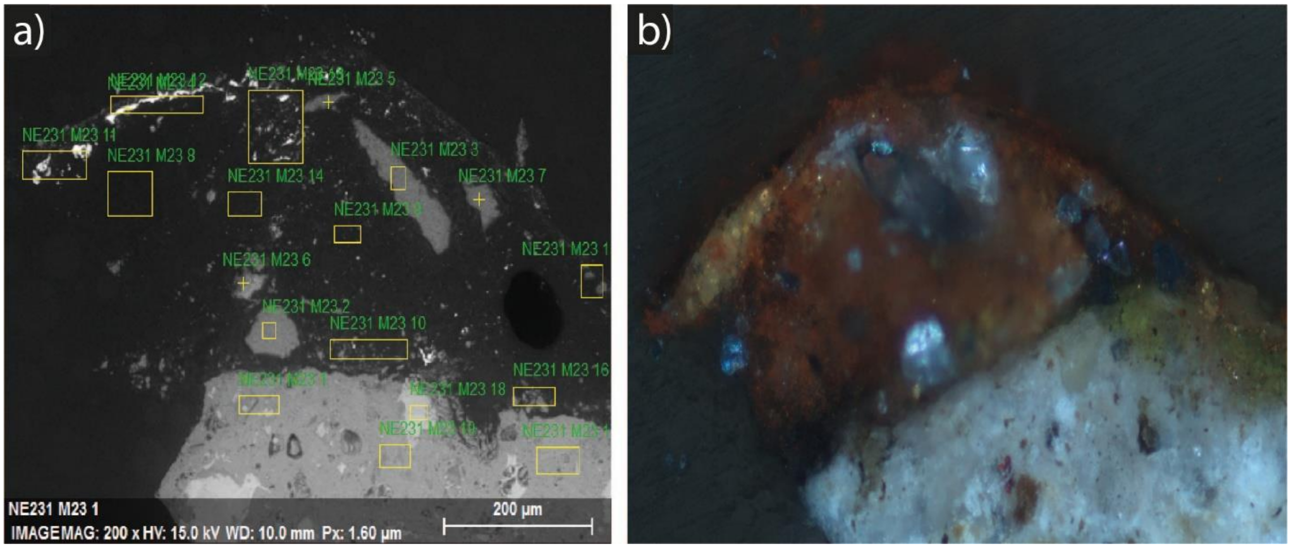

[illegible]

[illegible]

Spectrum	Mass percent (%)											
	C	O	Na	Mg	Al	Si	Ca	Fe	Cu	Zn	Ag	Au	Pb
NE231 M23 1	59.67	47.83	0.30	0.55	1.51	7.17	16.07	0.87					
NE231 M23 2	87.86	20.90			0.32	28.47	3.92						
NE231 M23 3	90.43	19.97	0.09		0.21	27.04	1.79						
NE231 M23 4	47.61	12.51				0.84	1.27				1.48	20.04	
NE231 M23 5	91.56	18.74				23.02	1.32						
NE231 M23 6	64.34	36.22		0.11	0.65	4.23	13.92	1.41					
NE231 M23 7	101.13	23.73			0.24	28.14	2.09						
NE231 M23 8	72.42	25.82			0.07	0.48	1.21						
NE231 M23 9	68.88	25.99			0.17	1.87	2.47	0.62					
NE231 M23 10	62.74	28.62		0.04	0.28	1.68	6.52						
NE231 M23 11	67.76	24.97			0.38	1.40	1.31	0.75				3.44	
NE231 M23 12	67.54	25.17	0.30		0.09	1.12	1.14	1.18			0.20	3.26	
NE231 M23 13	68.25	24.06	0.20		0.45	2.16	1.81	0.93				2.14	
NE231 M23 14	70.39	25.53			0.18	1.12	2.10	0.68					
NE231 M23 15	64.75	27.83			0.24	4.57	2.07	0.53					
NE231 M23 16	81.44	40.16		0.05	0.50	2.24	10.95	0.62					
NE231 M23 17	45.20	42.14		0.12	1.05	3.48	17.91	0.57					
NE231 M23 18	47.09	39.12		0.31	2.25	4.50	22.02	0.84					
NE231 M23 19	50.66	47.34		0.21	1.84	4.47	19.12	0.85					

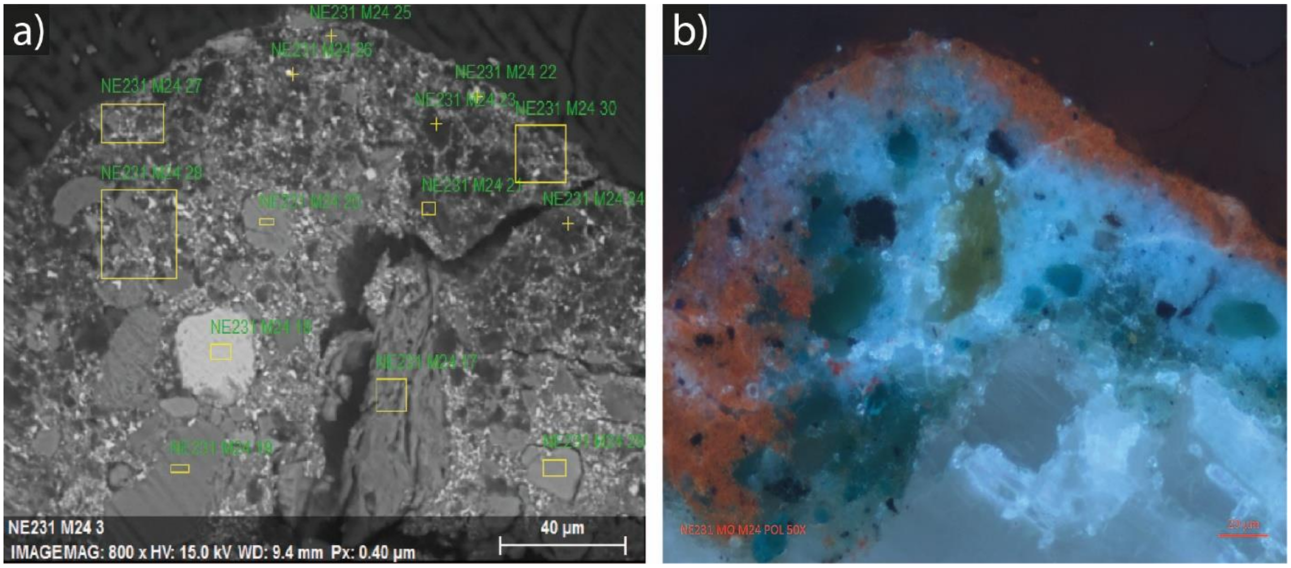

[illegible]

[illegible]

Spectrum	Mass percent (%)											
	C	O	Mg	Al	Si	S	K	Ca	Ti	Cr	Fe	Zn	Cd
NE231 M24 17	54.40	38.44	0.83	1.00	7.44	0.33	1.16	5.74	0.30		5.91	3.10	
NE231 M24 18	65.28	22.11	0.22	0.29	1.78	17.20	0.24	3.14			13.29	2.97	
NE231 M24 19	53.55	39.60	1.04	1.11	9.38	0.56	2.05	4.20			6.64	2.85	
NE231 M24 20	54.18	39.95	0.21	0.28	1.79	0.35	0.31	3.60		14.32	1.45	5.35	
NE231 M24 21	81.70	33.23	0.28	0.34	1.70	0.21	1.16	3.47		0.38	1.59	5.96	
NE231 M24 22	56.68	31.56		0.16	1.04	0.29		2.32			23.53	2.92	
NE231 M24 23	66.73	27.20	0.09	0.21	1.38	0.20	0.31	2.81			2.01	4.53	
NE231 M24 24	75.16	33.15		0.38	1.57	2.28		4.69		0.68	1.63	3.86	
NE231 M24 25	77.29	24.67		0.31	19.26	0.23	0.27	2.96			1.64	2.45	
NE231 M24 26	53.58	22.42	0.09	0.17	1.82	1.47		2.81		0.36	3.27	5.87	5.34
NE231 M24 27	66.90	33.43	0.23	0.39	2.49	0.31	0.58	4.03		0.43	2.94	10.52	
NE231 M24 28	62.46	34.39	0.39	0.51	3.44	0.37	0.74	6.04		0.46	2.66	9.52	
NE231 M24 29	48.73	41.98	0.25	0.25	1.60	0.13	0.26	4.52		15.63	1.23	3.52	
NE231 M24 30	67.46	31.17	0.16	0.32	2.18	0.78	0.21	3.63		0.39	6.38	4.49	

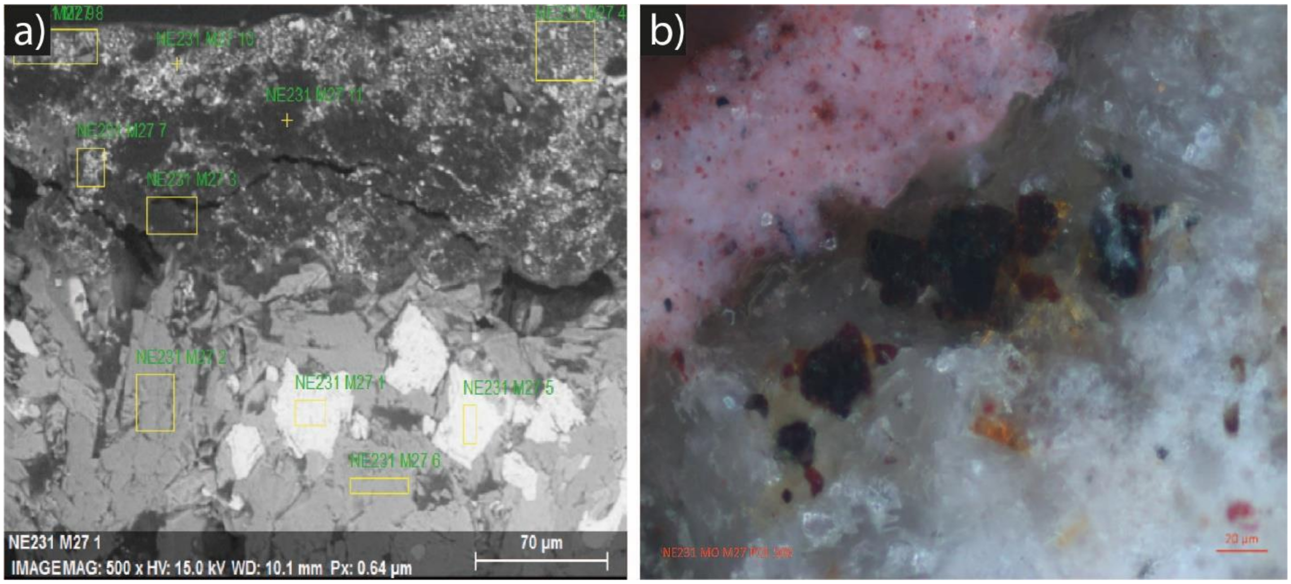

[illegible]

[illegible]

Spectrum	Mass percent (%)										
	C	O	Na	Mg	Al	Si	K	Ca	Ti	Fe	Zn
NE231 M27 1	44.34	38.26	0.98	1.56	1.77	3.99	0.58	2.52	1.03	19.31	1.09
NE231 M27 2	64.29	47.50	2.52		5.24	13.68	2.42	2.62		1.37	1.04
NE231 M27 3	65.63	32.12	1.08	0.17	0.94	2.73	0.39	1.75		1.30	3.41
NE231 M27 4	66.58	31.06	1.97		0.51	1.51	0.21	1.89		3.52	10.56
NE231 M27 5	47.78	42.43	0.98	1.72	1.82	4.18	0.58	3.01	0.81	23.96	1.50
NE231 M27 6	55.61	48.28	2.77	0.45	5.21	13.72	2.26	3.27		2.26	
NE231 M27 7	67.54	30.63	1.90	0.08	0.65	1.86	0.26	1.38		1.60	12.55
NE231 M27 8	64.66	31.37			0.71	2.08	0.21	1.03		4.00	8.90
NE231 M27 9	63.71	38.15	0.94		0.44	1.34		0.95		21.39	4.44
NE231 M27 10	72.67	31.32			0.59	1.73		1.16		1.06	21.53
NE231 M27 11	66.68	27.61	1.37		0.58	1.73		1.49		1.16	6.12

[illegible]

Microsample	OM	OMUV-FITC	μER-FTIR 1321 cm^{-1}	SEM	EDS
M8	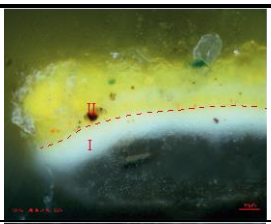	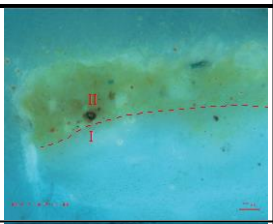	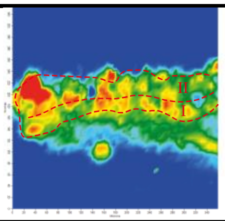	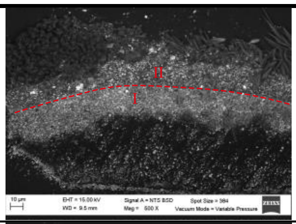	II: C, O, Mg, Si, Cr, Zn, Cd, Pb<br>I: C, O, Zn
M11	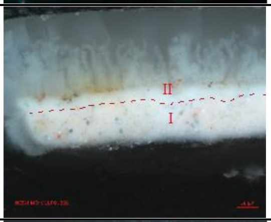	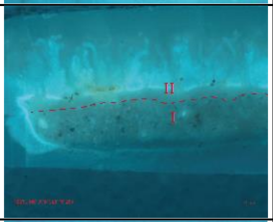	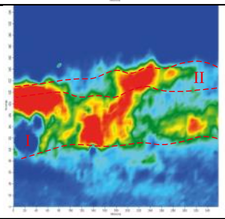	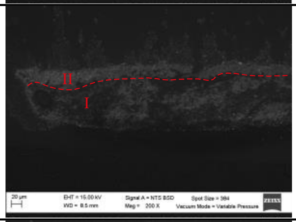	II:C, O, Ti, Zn<br>I: C, O, Si, Zn
M14	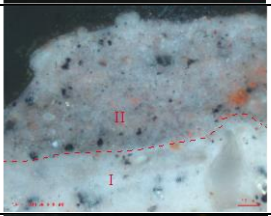	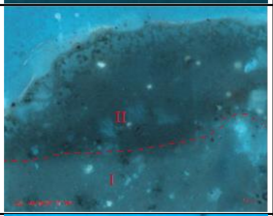	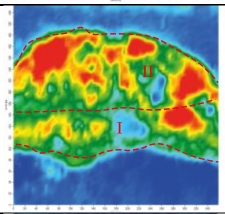	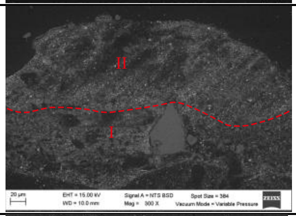	II:C, O, Mg, Al, Si, Ca, Fe, Zn, Mo<br>I:C, O, Al, Si, Ca, Zn
M20	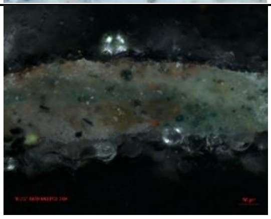	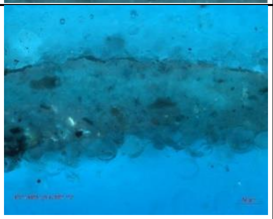	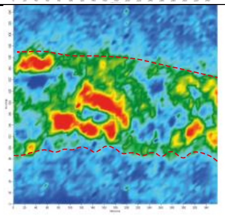	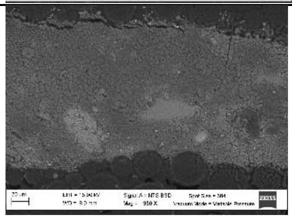	C, O, Mg, Al, Si, S, Ca, Ti, Cr, Fe, Zn, Cd
M23	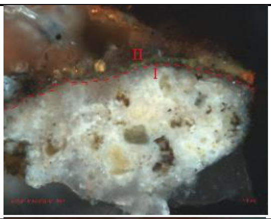	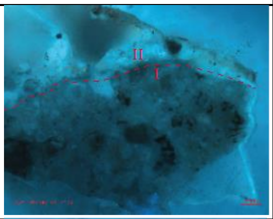	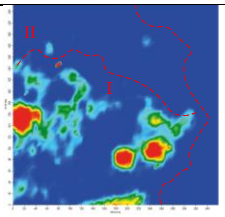	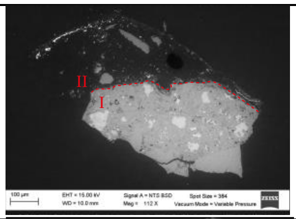	II: C, O, Al, Si, Ca, Fe, Cu, Zn, Ag, Au, Pb<br>I: C, O, Mg, Al, Si, Ca, Fe
M24	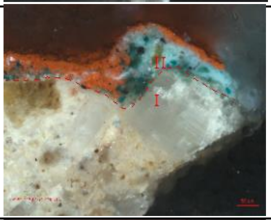	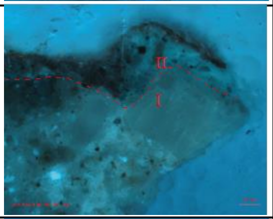	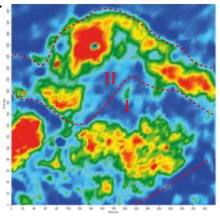	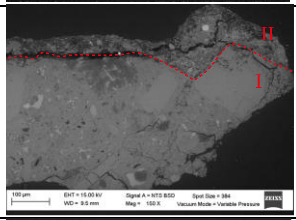	I:C, O, Mg, Al, Si, K, Ca, Ti, Cr, Fe, Zn, Ba, Cd<br>II:C, O, Mg, Al, Si, S, K, Ca, Fe
M27	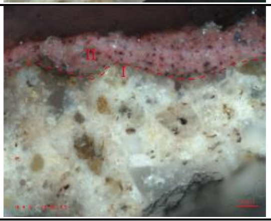	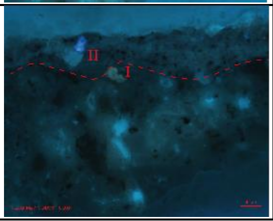	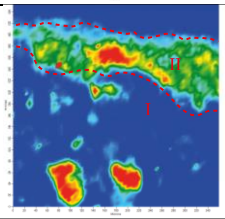	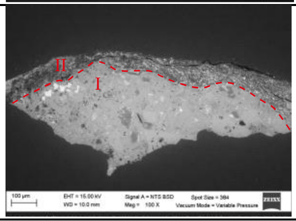	II: C, O, Al, Si, Ca, Fe, Zn<br>I:C, O, Mg, Al, Si, Ca, Ti, Fe, Zn

[illegible]

[illegible]

[illegible]

[illegible]

[illegible]

[illegible]

[illegible]
